# LIN28B induced PCAT5 promotes endometrial cancer progression and glycolysis via IGF2BP3 deubiquitination

**DOI:** 10.1038/s41419-024-06564-2

**Published:** 2024-04-02

**Authors:** Bin Wang, Bo Wang, Jian Ma, Jun-jian He, Zi-hao Wang, Qing Li, Xiao-xin Ma

**Affiliations:** Department of Obstetrics and Gynecology, Shengjing Hospital of China Medical University, 39 Huaxiang Road, Tiexi District, Shenyang City, Liaoning Province 110022 China

**Keywords:** Endometrial cancer, Cancer metabolism

## Abstract

Endometrial cancer (EC) cells exhibit abnormal glucose metabolism, characterized by increased aerobic glycolysis and decreased oxidative phosphorylation. Targeting cellular glucose metabolism in these cells could be an effective therapeutic approach for EC. This study aimed to assess the roles of LIN28B, PCAT5, and IGF2BP3 in the glucose metabolism, proliferation, migration, and invasion of EC cells. LIN28B highly expressed in EC, binds and stabilizes PCAT5. PCAT5, overexpressed in EC, and its 1485-2288nt region can bind to the KH1-2 domain of IGF2BP3 to prevent MKRN2 from binding to the K294 ubiquitination site of IGF2BP3, thus stabilizing IGF2BP3. Finally, IGF2BP3 promotes the aerobic glycolysis, proliferation, migration and invasion of EC cells by stabilizing the key enzymes of glucose metabolism HK2 and PKM2. Taken together, our data reveal that the LIN28B/PCAT5/IGF2BP3 axis is critical for glucose reprogramming and malignant biological behavior in EC cells. Therefore, targeting this axis may contribute to the development of a novel therapeutic strategy for EC metabolism.

## Introduction

At the present, endometrial cancer (EC) is responsible for approximately annual 76,000 mortalities among women globally, posing a significant threat, particularly in developed countries, where its morbidity and mortality rates are rising [1]. Despite advancements in targeted and immunotherapy benefiting some patients, those with advanced EC continue to face a poor prognosis. The genetic and epigenetic modifications driving EC development and influencing treatment response remain largely unexplored [2]. In the late 1920s, Otto Warburg discovered that cancer cells still have a high rate of glycolysis and lactic acid production even when oxygen is abundant, which is called the Warburg effect or aerobic glycolysis [3]. A number of studies have found that aerobic glycolysis is closely related to the development of EC [4–6]. But there is still a region for further research.

LIN28B, a highly conserved RNA-binding protein (RBP) in higher eukaryotes, functions as an oncogene involved in the progression of various cancers [7–9]. Recent studies have also identified its role in promoting malignant behaviors in EC [10, 11]. Moreover, LIN28B has been linked to the promotion of aerobic glucose metabolism in tumors [12, 13]. However, the relationship between LIN28B and aerobic glycolysis in EC has not been reported.

Long noncoding RNA (lncRNA) is a kind of conserved noncoding RNA with more than 200 nucleotides in length that does not have any protein-coding ability. Abnormal expression of lncRNA exists in many types of cancer and plays an important regulatory role in a variety of complex biological processes such as cell proliferation and differentiation [14]. In recent years, researchers have found that lncRNAs can regulate cell energy metabolism and reprogramming by mediating post-translational modification in a variety of cancers, including EC [5, 15]. LncRNA-PCAT5 is a new oncogenic lncRNA in ERG-positive prostate cancer, which has the prospect of becoming a biomarker of prostate cancer and a new therapeutic target [16]. However, its role in the EC is still unclear.

IGF2BP3 is a RBP that has attracted much attention. It has the ability to stabilize mRNA in eukaryote [17], and it has been found to have extensive regulatory ability in a variety of cancers as an oncogene RBP [18, 19]. In particular, it was found to be closely associated with clinicopathological parameters of EC [20, 21]. In lung cancer, IGF2BP3 has been reported to have the ability to regulate the alternative splicing of Pyruvate kinase M (PKM), suggesting that IGF2BP3 may play a role in regulating glucose metabolism reprogramming [22]. In addition, IGF2BP3 has been found to be frequently post-transcriptionally regulated [23, 24]. However, it is unknown whether IGF2BP3 is post-transcriptionally regulated in EC or is involved in glucose metabolism.

Hexokinase 2 (HK2), a glycolytic enzyme that catalyzes the first step of glucose metabolism, can be significantly induced in tumor cells through a variety of mechanisms [3, 25]. HK2 was highly expressed in EC tissues, and its expression was associated with poor overall survival. HK2 overexpression can effectively promote the epithelial–mesenchymal transition (EMT) phenotype and enhance aerobic glycolysis of EC cells by activating FAK and its downstream ERK1/2 signaling pathway [4]. Pyruvate kinase M2 (PKM2) is a key rate-limiting enzyme in glycolysis. It can catalyze the phosphorylation of pyruvate phosphate and adenosine diphosphate to produce pyruvate and ATP. It is also a key regulator of tumor metabolism [26]. High expression of PKM2 was also found to be significantly associated with EC and worse prognostic factors [27, 28]. It is unknown if IGF2BP3 influences the expression of HK2 and PKM2 in EC and so affects aerobic glycolysis. In this study, we found that highly expressed LIN28B can stabilize PCAT5. PCAT5 can prevents Makorin ring finger protein 2 (MKRN2) induced ubiquitination degradation by binding to the K294 site of IGF2BP3. Then, IGF2BP3 can stabilizes the mRNA of HK2 and PKM2, and promotes the aerobic glycolysis, proliferation, migration, and invasion of EC.

## Materials and methods

### Human tissue specimens

A total of 50 EC tissues and 49 normal endometrial tissues were collected from patients undergoing surgery in the Department of Obstetrics and Gynecology at Shengjing Hospital of China Medical University from 2017 to 2020. None of the patients had received preoperative radiotherapy or chemotherapy. The diagnosis of EC was determined by histopathological observation of postoperative paraffin sections according to the staging standard of the International Federation of Obstetrics and Gynecology (FIGO 2009). All patients were provided with informed consent. This study was approved by the ethics committee of Shengjing Hospital of China Medical University (2020PS270K).

### Cell culture

The Ishikawa cell line and HEC-1A cell line were maintained in RPMI 1640 medium (Gibco, Carlsbad, CA, USA) or McCoy S 5 A medium (Gibco), respectively. The human embryonic kidney cell line HEK293T was maintained in high-glucose Dulbecco’s Modified Eagle Medium (Gibco). Cells were cultured in the medium containing 10% fetal bovine serum (FBS) (Gibco). All cells were cultured in 37 °C humidified incubators with 5% of CO_2_. The cells were obtained from the Institute of Biochemistry and Cell Biology (All cell lines were identified by STR), Chinese Academy of Sciences (Shanghai, China).

### Cell transfection

We constructed lentiviral plasmids that overexpression or knockdown of LIN28B, PCAT5, IGF2BP3 and corresponding to negative control (NC) in Genechem (Shanghai, China). The lentivirus plasmid that knocks down MKRN2 and the corresponding NC was constructed. ShRNA targeting sequences of LIN28B, PCAT5, IGF2BP3, and MKRN2 are shown in Table S1. Lentiviral transfection was performed according to the instructions. The results of overexpression and knockdown efficiency of LIN28B, PCAT5, IGF2BP3 were shown in Fig S1A–C. In addition, we constructed full-length or segmented IGF2BP3 plasmids labeled with FLAG and full-length MKRN2 labeled with HA by Genepharma (Shanghai, China). IGF2BP3-K294-mut was generated by site-directed mutagenesis via Quik Change Lightning Site Directed mutagenesis kit (210518, Agilent Technologies Inc., USA).

### RNA extraction and real-time fluorescence quantitative PCR

Total RNA was extracted from tissues or cells using Trizol reagent (Takara, Dalian, China). The expression of target gene mRNA was detected by the 7500 fast real-time polymerase chain reaction (PCR) system (Applied Biosystems, USA) using a one-step SYBR primescript RT-PCR Kit (RR064A, Takara, Dalian, China). β-actin was used as an endogenous control gene, and the primers used in the study are shown in Table S1. The multiple changes were calculated using relative quantification (2-^∆∆^Ct).

### Western blot

We used RIPA lysate (P0013B, Beyotime, China) to extract total proteins from tissues or cells. Protein samples were transferred to a polyvinylidene fluoride (PVDF) membrane by sodium dodecyl sulfate-polyacrylamide gel electrophoresis (SDS-PAGE). Then, the membrane was incubated in 5% skim milk for 2 h, and the primary antibody was incubated at 4 °C overnight. The membranes were incubated with appropriate horseradish peroxidase coupled with secondary antibodies for 2 h at room temperature. Finally, an enhanced chemiluminescence (ECL) kit (P0018AS, Beyotime, China) was used for chemiluminescence under Image Lab software (Beta 3.0, Bio-rad, California, USA). Using β-actin as an endogenous control, relative integral density (IDV) was calculated by image-J software. The antibodies used in the experiment are shown in Table S2.

### Immunohistochemistry

We selected paraffin samples from clinical samples (EC or normal endometrium) or xenograft tumors from nude mice for immunohistochemistry (IHC). First, the slices were dewaxed. Then the slices were placed in Tris-Ethylenediaminetetraacetic acid (EDTA) (pH 9.0, Solarbio, Beijing, China) and bathed in water at 100 °C for 10 min, then re-warmed at room temperature for 30 min. We selected the UltraSensitive^TM^ SP (mouse or rabbit) IHC Kit (kit-9710, Maixin, Fuzhou, China) to block the endogenous peroxidase of the sample and block it with the non-specific staining. The 3,3’-diaminobenzidine (DAB) kit (DAB-0031, Maixin, Fuzhou, China) was used for color rendering. The area was quantified by an optical microscope. The antibodies used in the experiment are shown in Table S2.

### Fluorescence in Situ Hybridization (FISH) and immunofluorescence (IF)

In order to verify the localization of PCAT5 with LIN28B or IGF2BP3 in cells, we constructed a FISH probe for PCAT5 in Genepharma (Shanghai, China) (Table S1). Perform the FISH experiment according to the instructions. The slices were incubated with Cy3at 37 °C for 60 min. The nuclei were stained with DAPI (Beyotime, China). A fluorescence microscope (C1002, Nikon LumiCite 6000, Japan) was used to take photos, and image-J was used to analyze the images. The antibodies used in the experiment are shown in Table S2.

### LncRNA microarray

We used stable knockdown of LIN28B and the corresponding NC Ishikawa and HEC-1A cells as the test samples. The total RNA was extracted from each sample according to the manufacturer’s protocol, and the concentration and quality of extracted RNA were evaluated by a nanodrop spectrophotometer (Nd-100, Thermo, USA). The RNA was amplified and transcribed into cDNA using the rtStar First-Strand cDNA Synthesis Kit (AS-FS-001, Aksomics, Shanghai, China). Finally, the samples were tested by lncRNA microarray (Aksomics, Shanghai, China) (Table S3).

### RNA immunoprecipitation (RIP) assay

RIP was determined using the Magna RIP^TM^ RNA-Binding Protein Immunoprecipitation Kit (17-700, Millipore, USA) in accordance with the manufacturer’s protocols, with 5 μg LIN28B IGF2BP3 or FLAG antibody (Table S2), and IgG as a NC. First, the cell lysate was incubated with a complex of RIP buffer magnetic beads and antibodies. The complex was then incubated with protease K to isolate the immunoprecipitated RNA. Finally, the purified RNA was analyzed by quantitative reverse transcription–PCR (RT-qPCR) and agarose gel electrophoresis to prove the existence of binding targets.

### RNA-Pulldown and Liquid chromatography–mass spectrometry (LC–MS)

In order to verify the protein combined with PCAT5, we constructed the RNA-pulldown probe of sense and antisense biotin-labeled PCAT5 in Genechem (Shanghai, China; Table S1). In short, according to the instructions, the Pierce^TM^ Magnetic RNA-Protein Pull-down Kit (20164, Thermo Scientific, USA) was used. HEK293T cell lysates with stable overexpression of PCAT5 were co-incubated with probes for 4 h. Then, the probe-protein conjugate was incubated with streptavidin magnetic beads overnight. The RNA-protein-magnetic bead mixture was collected in a centrifuge and eluted on a Handee rotating column. The mixture was washed briefly three times and boiled in SDS buffer. The samples were then subjected to SDS-PAGE. Rapid silver staining (P0017S, Beyotime, China) was used for protein coloration. After the differential bands were found, the cut differential bands were sent to BGI Genomics (Shenzhen, China) for LC–MS identification of proteins (Table S4).

### Immunoprecipitation (IP)

FLAG-labeled full-length or fragmented IGF2BP3 plasmid or HA labeled full-length MKRN2 plasmid were transfected into HEK293T cells. Then, the collected cells were fully lysed in RIPA buffer containing protease inhibitor and ribonuclease inhibitor and centrifuged at 4 °C, 17,000 g for 15 min. Incubate the cell lysates with 5 g of the corresponding antibody (Table S2) and Protein A/G magnetic beads overnight at 4 °C, rotating gently. Wash three times with NT2 buffer (50 mM Tris-HCl, pH 7.4, 150 mM NaCl, 0.05% Nonidet P-40, 1 mM MgCl2). Wash once more with PBS containing protease and ribonuclease inhibitors. Then heat it at 95 °C for 7 min, and then use the corresponding antibody (Table S2) for Western blot detection.

### Nascent RNA capture

We used the clickit RNA Capture Kit (C10365, Invitrogen, Carlsbad, CA, USA) in EC cells to capture nascent RNA. The nascent RNA was labeled biotinylated with 5-ethyluridine (EU), and the labeled RNA was separated by magnetic streptavidin beads for subsequent RT-qPCR.

### RNA stability measurement

Actinomycin D (HY-17559, MCE, Shanghai, China) was added into the culture medium for EC cells to inhibit cell transcription. Cells were collected at 0, 2, 4, 6, 8, and 10 h after actinomycin D was added. The expression level of RNA was detected by RT-qPCR. Compared with 0 h, the half-life was measured when the RNA expression reached 50%.

### Measurement of extra cellular acidification rate (ECAR)

ECAR was measured using the seahorse XF glycolysis pressure test kit (103020-100, seahorse Bioscience, USA). In brief, the stable transfection EC cells were cultured in a 96 well XF microplate (102416-100, Seahorse Biosciences, USA) with 2 × 10^4^ per well and cultured overnight at 37 °C under 5% CO_2_. The cells were incubated at 37 °C in 150 ul medium for 1 h. After calibrating the probe, glucose (5 µM) oligomycin (1.5 µM) and 2-DG (5 µM) were injected into A, B, and C, respectively. Data were evaluated and analyzed using a Seahorse XF96 analyzer.

### Measurement of glucose consumption and lactate concentration

We used the glucose uptake kit (AB136955, Abcam, USA) and the lactate assay kit (Nanjing, China) for detection. EC cells at 1.5 µl per well 10^5^ cells were inoculated in 96-well plates containing 200 µl of medium. After continued culture for 24–48 h, the supernatant culture medium was collected and the glucose consumption and lactate concentration were detected by a colorimetric method according to the manual.

### Cell proliferation assay

We used the EdU cell proliferation kit (CX003, Cellorlab, Shanghai, China) to measure the cell proliferation rate. According to the manufacturer’s instructions, add 20 µM EdU reagent to the selected EC cell culture medium and incubate for 2 h. After the cells were fixed with 4% paraformaldehyde, DNA staining was performed, then cell staining was added and the cells were washed with PBS. Fluorescence microscopy (C1002, Nikon LumiCite 6000, Japan) was used to take photos. Image-j was used to fuse the images, and the proliferation rate was calculated.

### Wound-healing

We used a wound-healing assay to evaluate cell migration ability. The selected EC cells were inoculated into six-well plates. When the growth reached about 90% confluence, a wound was artificially established on the cell monolith with the tip of a 100 µl pipette. After the cells were cleaned with PBS, the cells were cultured in fresh serum-free medium. The capacity of cells to migrate to the wound site was monitored daily with a microscope and photographed at 0 and 48 h with a light microscope.

### Transwell assay

We used the transwell assay to evaluate the ability of cell invasion. Pre-apply the matrix glue to the upper chamber (aperture 8 mm; Corning, NY, USA), then the selected cells are suspended in 200 µl of serum-free medium and placed in the upper chamber. In addition, fill the lower chamber with 800 µl of medium containing 10% FBS. Several hours later, some suspended cells were observed in the lower chamber, and the chamber pores were cleaned. The cells were stained with Giemsa staining solution (Leagene Biotechnology, Beijing, China). The non-invasive cells on the surface of the upper chamber were wiped with a cotton swab. Images were obtained using an inverted microscope and analyzed.

### Cycloheximide and MG132

The protein synthesis inhibitor cycloheximide (CHX) (50 μg/mL) was added to the cell culture medium and incubated for 0–12 h. After total proteins were collected, western blotting was performed. The proteasome inhibitor MG132 (25 μm) was added to the cell culture medium and incubated for 8 h. Total proteins were collected and western blotting was performed.

### Xenograft tumor experiment in nude mice

In this in vivo study, female four-week-old nude mice without thymus (BALB/ C) were purchased from Huafukang Biotechnology Co., Ltd., (Beijing, China). All animal experiments are carried out in accordance with the Animal Welfare Law and approved by the Ethics Committee of China Medical University (2020PS190K). Nude mice were randomly divided into five groups: control group, LIN28B (−) group, PCAT5 (−) group, IGF2BP3 (−) group, and LIN28B (−) + PCAT5 (−) + IGF2BP3 (−) group. In the rescue experiment we randomly divided the nude mice into six groups: control group, LIN28B(−) + PCAT5( + )NC group, LIN28B (−) + PCAT5(+) group, control group, PCAT5(−) + IGF2BP3( + )NC group, PCAT5(−) + IGF2BP3(+)group. During subcutaneous implantation, 3 × 10^5^ stably transfected and expressed cells were injected subcutaneously into the right armpit.

### Statistical analysis

Data were presented as mean ± standard deviation (SD) of three independent experiments. Graphpad Prism 8 software (Graphpad, San Diego, CA, USA) was used for statistical analysis. The normality of the data was analyzed by the D’Agostino, Pearson, and Shapiro Wilk tests. A non-paired two-sided t-test was used for data with a normal distribution, and a nonparametric Mann Whitney test was used to compare the two groups. One-way ANOVA was used to compare the differences among more than two groups. A Pearson or Spearman’s rank correlation coefficient analysis is used to detect correlation. The data is presented as a mean ± standard deviation. *P* < 0.05 was statistically significant.

## Results

### High expression of LIN28B is associated with poor prognosis of EC patients, and knockdown of LIN28B can inhibit glycolysis, proliferation, migration, and invasion of EC cells

**Fig. 1:**
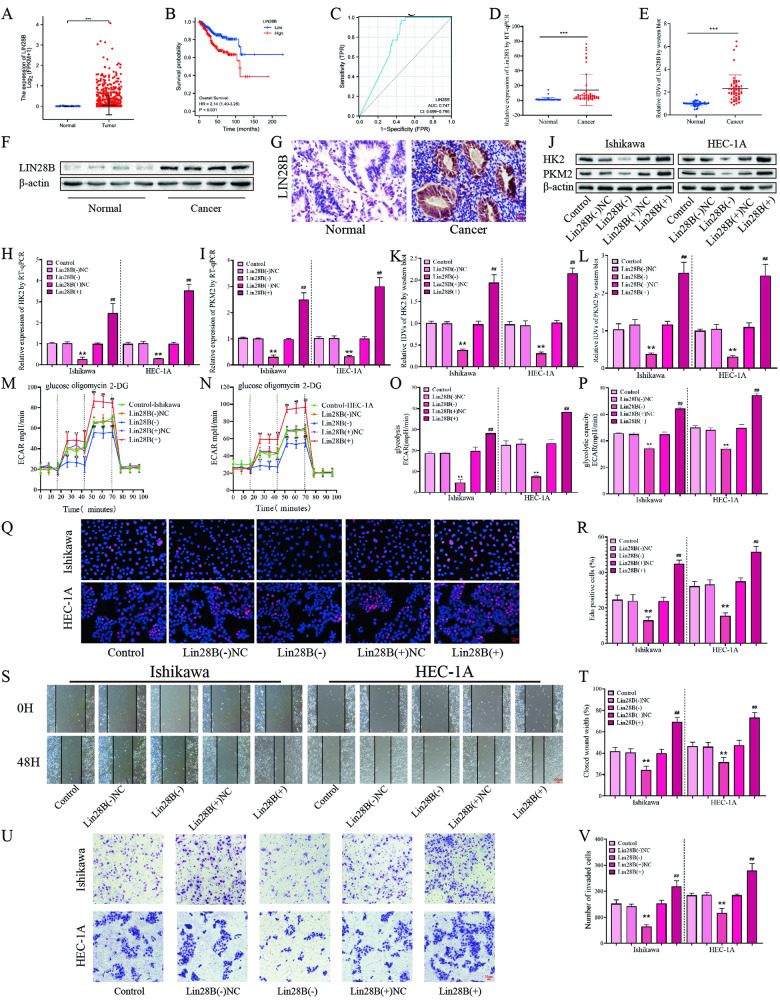
The expression of LIN28B and its efects on glycolysis, proliferation migration and invasion in EC. **A** Expression of LIN28B in TCGA cohort of endometrial tissues, tumor (n = 552), compared with normal endometrial tissue (*n* = 35). **B** Effect of LIN28B expression level on EC patient survival time from TCGA database. **C** ROC of LIN28B (TCGA cohort). **D** The *LIN28B* expression levels in the EC (*n* = 50) and control (*n* = 49) group were detected by RT-qPCR. **E**, **F** The protein expression levels of LIN28B in the EC (*n* = 50) and control (*n* = 49) group were detected by western blot. **G** The expression of LIN28B was detected via immunohistochemistry in EC (*n* = 50) and control (*n* = 49), Scar bar = 25 μm. **H**, **I** The effects of LIN28B overexpression or silencing on the HK2 and PKM2 expression in Ishikawa and HEC-1A cells was assessed by RT-qPCR (*n* = 3). **J**–**L** The effects of LIN28B overexpression or silencing on the HK2 and PKM2 expression in Ishikawa and HEC-1A cells was assessed by western blot (*n* = 3). **M**–**P** The effects of LIN28B overexpression or silencing on the glycolysis ability in Ishikawa and HEC-1A cells was assessed by ECAR (*n* = 3). **Q**, **R** The effects of LIN28B overexpression or silencing on the proliferation ability in Ishikawa and HEC-1A cells was assessed by EdU assay (*n* = 3), Scar bar = 50 μm. **S**, **T** The effects of LIN28B overexpression or silencing on the migration ability in Ishikawa and HEC-1A cells was assessed by wound-healing assay (*n* = 3), Scar bar = 100 μm. **U**, **V** The effects of LIN28B overexpression or silencing on the transwell ability in Ishikawa and HEC-1A cells was assessed by transwell assay (n = 3), Scar bar = 50μm. Data are expressed as means ± standard deviation. *p < 0.05; **p < 0.01; ***p < 0.001.

In order to verify the abnormal expression of LIN28B in EC patients, we used the TCGA database (https://portal.gdc.cancer.gov/), and found that its expression in patients with endometrial cancer (*n* = 552) was significantly higher than that in the control group (*n* = 35) (Fig. 1A). High expression of LIN28B was correlated with worse overall survival of EC patients (Fig. 1B). In particular, LIN28B was found to have high specificity and sensitivity for EC diagnosis (Fig. 1C). The transcription and expression of LIN28B in EC tissues were significantly increased in our study group (Fig. 1D–F). IHC experiments showed that LIN28B was mainly expressed in the cytoplasm of EC tissues, and the expression level was significantly higher than that of the control group (Fig. 1G, Tables S5). In addition, we analyzed the mRNA and protein expression levels of LIN28B and clinicopathological parameters of EC patients. The results showed that LIN28B expression was significantly correlated with clinicopathological features. Notably, LIN28B expression increased significantly as the clinical stage progressed from I + II to III + IV and significantly increased in patients with lymph node metastasis (Tables S6, S7).

In order to study the effects of LIN28B on glycolysis, proliferation, migration, and invasion of EC. First, we established Ishikawa and HEC-1A cell lines with stable overexpression or knockdown of LIN28B using lentivirus as vectors. RT-qPCR experiments showed that overexpression of LIN28B could significantly improve the transcription of HK2 and PKM2 (Fig. 1H, I). Overexpression of LIN28B can significantly improve the expression of HK2 and PKM2 as demonstrated by western-blot experiments (Fig. 1J–L). Though extra cellular acidification rate (ECAR) experiments showed that overexpression of LIN28B could increase glycolysis and glycolytic capacity in EC cells, the knockdown of LIN28B had the opposite effect (Fig. 1M–1P). In addition, the glucose consumption and lactate concentration assays were significantly increased after LIN28B overexpression (Fig. S2A, B). These results suggest that LIN28B can promote aerobic glycolysis in EC. Additionally, the EdU experiment found that overexpression of LIN28B significantly promoted proliferation in both cell lines (Fig. 1Q, R). Finally, the wound-healing and transwell assays showed that overexpression of LIN28B promoted migration and invasion ability in EC cell lines, while knockdown of LIN28B significantly reduced its migration and invasion ability (Fig. 1S–V).

### Knockdown of PCAT5 can inhibit glycolysis, proliferation, migration, and invasion of EC cells

**Fig. 2:**
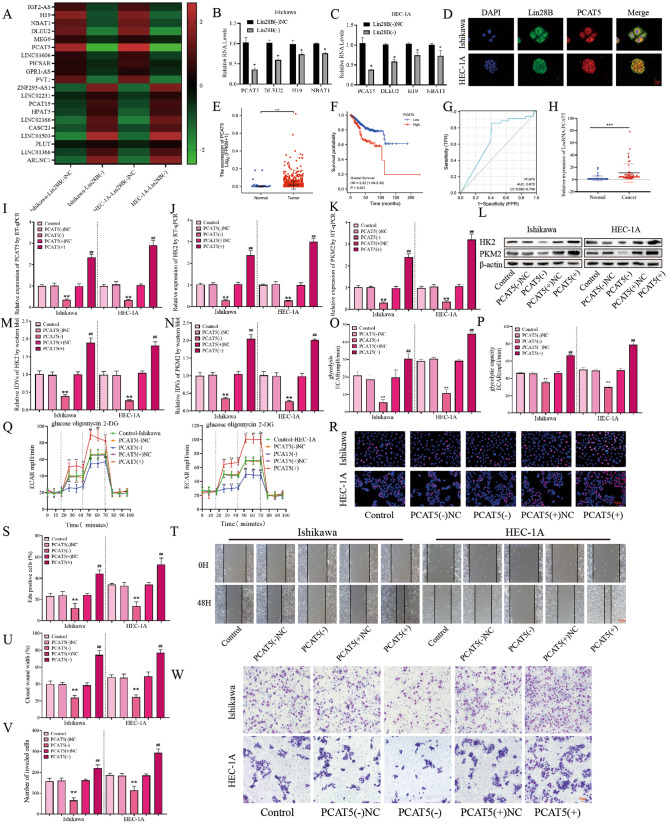
The expression of PCAT5 and its efects on glycolysis, proliferation migration and invasion in EC. **A** lncRNA microarray was performed to detect the gene profile when LIN28B was knockdown in the EC cells (*n* = 3 per group). **B**, **C** RT-qPCR was performed to detect the lncRNA expression of PCAT5, DLEU2, H19 and NBAT1 when LIN28B was knockdown in the EC cells (*n* = 3). **D** FISH and IF double staining was performed to detected the binding effects between LIN28B and PCAT5 in EC cells (*n* = 3) Scar bar = 25 μm. **E** Expression of PCAT5 in TCGA cohort of endometrial tissues, tumor (*n* = 552), compared with normal endometrial tissue (*n* = 35). **F** Effect of PCAT5 expression level on EC patient survival time from TCGA database. **G** ROC of PCAT5 (TCGA cohort). **H** The PCAT5 expression levels in the EC (*n* = 50) and control (*n* = 49) group were detected by RT-qPCR. **I**–**K** The effects of PCAT5 overexpression or silencing on the HK2 and PKM2 expression in EC cells was assessed by RT-qPCR (*n* = 3). **L**–**N** The effects of PCAT5 overexpression or silencing on the HK2 and PKM2 expression in EC cells was assessed by western blot (*n* = 3). **O**–**Q** The effects of PCAT5 overexpression or silencing on the glycolysis ability in EC cells was assessed by ECAR (*n* = 3). **R**, **S** The effects of PCAT5 overexpression or silencing on the proliferation ability in EC cells was assessed by EdU assay (*n* = 3), Scar bar = 50 μm. **T**, **U** The effects of PCAT5 overexpression or silencing on the migration ability in EC cells was assessed by wound-healing assay (*n* = 3), Scar bar = 100 μm. **V**, **W** The effects of PCAT5 overexpression or silencing on the transwell ability in EC cells was assessed by transwell assay (*n* = 3), Scar bar = 50 μm. Data are expressed as means ± standard deviation. *p < 0.05; **p < 0.01; ***p < 0.001.

In order to further explore the mechanism of LIN28B’s promoting EC glycolysis, proliferation, migration, and invasion, we established stable knockdown of LIN28B and NC in EC cells. Through lncRNA microarray and RT-qPCR experiments, it was found that lncRNA-PCAT5 decreased most significantly in LIN28B (−) cells (Fig. 2A–C; Table S3), which attracted our attention. Notably, the FISH + IF double staining experiment showed that LIN28B and PCAT5 could bind in the cytoplasm of EC cells (Fig. 2D). Then, we found that the expression of PCAT5 was significantly increased in EC from the TCGA database (Fig. 2E), and its high expression was correlated with worse overall survival of EC patients (Fig. 2F). In addition, PCAT5 has high accuracy in diagnosing EC (Fig. 2G). Further we found that PCAT5 expression in EC was significantly increased in our study group (Fig. 2H). In particular, we analyzed the clinicopathological parameters of patients with PCAT5 and EC and found that their expression was significantly correlated with clinicopathological features (Table S8). The expression of PCAT5 increased significantly with the progression of clinical stage from I + II to III + IV. Compared with the highly differentiated group, the expression of PCAT5 was significantly increased in the medium and low-differentiated groups. In addition, the high expression of PCAT5 was positively correlated with the degree of infiltration, lymph node, and distant metastasis (Table S8). These results suggest that PCAT5 has the potential as a biomarker in EC.

To study the effects of PCAT5 on EC glycolysis, proliferation, migration, and invasion, we established EC cell lines with stable overexpression and knockdown of PCAT5 through lentiviral vectors. Validation by RT-qPCR and western blot experiments showed that overexpression of PCAT5 significantly promoted the transcription and expression of HK2 and PKM2 in EC cells, while knockdown of PCAT5 had the opposite effect (Fig. 2I–2N). The validation by ECAR, glucose consumption, and lactate concentration assays showed that overexpression of PCAT5 could promote aerobic glycolysis in EC cells, while knockdown of PCAT5 could inhibit aerobic glycolysis (Fig. 2O–2Q, Fig S2C-2D). The overexpression of PCAT5 could significantly promote the proliferation of EC cells, and the knockdown of PCAT5 had the opposite effect (Fig. 2R–2S). The wound-healing and transwell assays showed that overexpression of PCAT5 promoted migration and invasion ability in EC cell lines, while knockdown of PCAT5 significantly reduced its migration and invasion ability (Fig. 2T–2W).

### LIN28B promotes glycolysis, proliferation, migration, and invasion of EC cells by stabilizing PCAT5

**Fig. 3:**
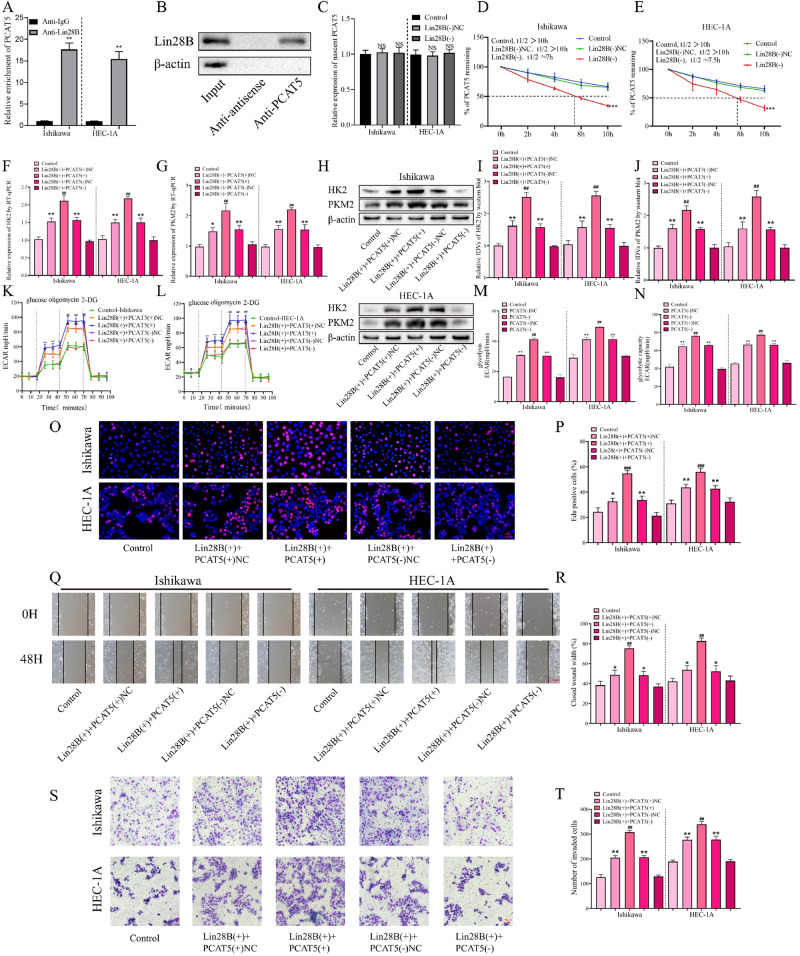
LIN28B promotes glycolysis, proliferation, migration and invasion of EC cells by stabilizing PCAT5. **A** An enrichment of PCAT5 in LIN28B immunoprecipitated samples via RNA immunoprecipitation (RIP) assay (*n* = 3). **B** RNA-pulldown assay followed by Western blot showed the specific associations of LIN28B with biotinylated-PCAT5 or antisense RNA (*n* = 3). **C** Expression of nascent PCAT5 was measured via RT-qPCR after LIN28B knockdown (*n* = 3). **D**, **E** Half-life of PCAT5 was measured by RT-qPCR after actinomycin D treated in EC cells (*n* = 3). **F**, **G** Regulation of HK2 and PKM2 transcription by LIN28B and PCAT5 was analyzed via RT-qPCR (*n* = 3). **H**–**J** Regulation of HK2 and PKM2 transcription by LIN28B and PCAT5 was analyzed via western blot (*n* = 3). **K**–**N** The effects of LIN28B and PCAT5 on the glycolysis ability in EC cells was assessed by ECAR (*n* = 3). **O**, **P** The effects of LIN28B and PCAT5 on the proliferation ability in EC cells was assessed by EdU assay (*n* = 3), Scar bar = 50 μm. **Q**, **R** The effects of LIN28B and PCAT5 on the migration ability in EC cells was assessed by wound-healing assay (*n* = 3), Scar bar = 100 μm. **S**, **T** The effects of LIN28B and PCAT5 on the transwell ability in EC cells was assessed by transwell assay (*n* = 3), Scar bar = 50μm. Data are expressed as means ± standard deviation. *p < 0.05; **p < 0.01; ***p < 0.001.

Our above results proved the carcinogenic effects of LIN28B and PCAT5 in EC, and the significantly reduced expression of PCAT5 in LIN28B (-) cells led us to speculate that there was a positive association between them. It is found that there is a positive correlation between the protein expression level of LIN28B and the expression level of PCAT5 in EC tissues by Spearman’s rank correlation coefficient analysis. (Fig S2E). Further validation by the RIP experiments showed that PCAT5 was significantly enriched in LIN28B compared with IgG (Fig. 3A). The RNA-pulldown assay further confirmed the binding mechanism between them (Fig. 3B). To further clarify the mechanism between LIN28B and PCAT5 in EC cells, we analyzed the effects of knockdown of LIN28B on nascent RNA and the half-life of PCAT5 by RT-qPCR. As shown in (Fig. 3C–3E), LIN28B has no significant effect on the nascent RNA of PCAT5, but can significantly reduce the half-life of PCAT5, suggesting that LIN28B has a stabilizing effect on PCAT5.

To investigate whether PCAT5 affects the mechanism of LIN28B’s ability to promote EC glycolysis, proliferation, migration, and invasion. PCAT5( + ), PCAT5(−), and their NC vectors were transfected into EC cells with LIN28B overexpression. It was found that PCAT5 knockdown could offset the promoting effect of LIN28B overexpression on the transcription and expression of HK2 and PKM2 (Fig. 3F–J). Similarly, we found by ECAR, glucose consumption, and lactate concentration assays that knockdown of PCAT5 could counteract the ability of overexpressing LIN28B to promote aerobic glycolysis of EC cells (Fig. 3K–N, Fig S2F, G). In addition, the EdU experiment found that knockdown of PCAT5 could counteract the promoting effect of LIN28B overexpression on the proliferation of EC cells (Fig. 3O, P). Finally, through wound-healing and transwell assays, it was found that knockdown of PCAT5 could offset the effect of overexpression of LIN28B on the migration and invasion ability of EC cells (Fig. 3Q–T).

### PCAT5 may bind directly to IGF2BP3 and inhibit its ubiquitin and proteasome-mediated degradation

**Fig. 4:**
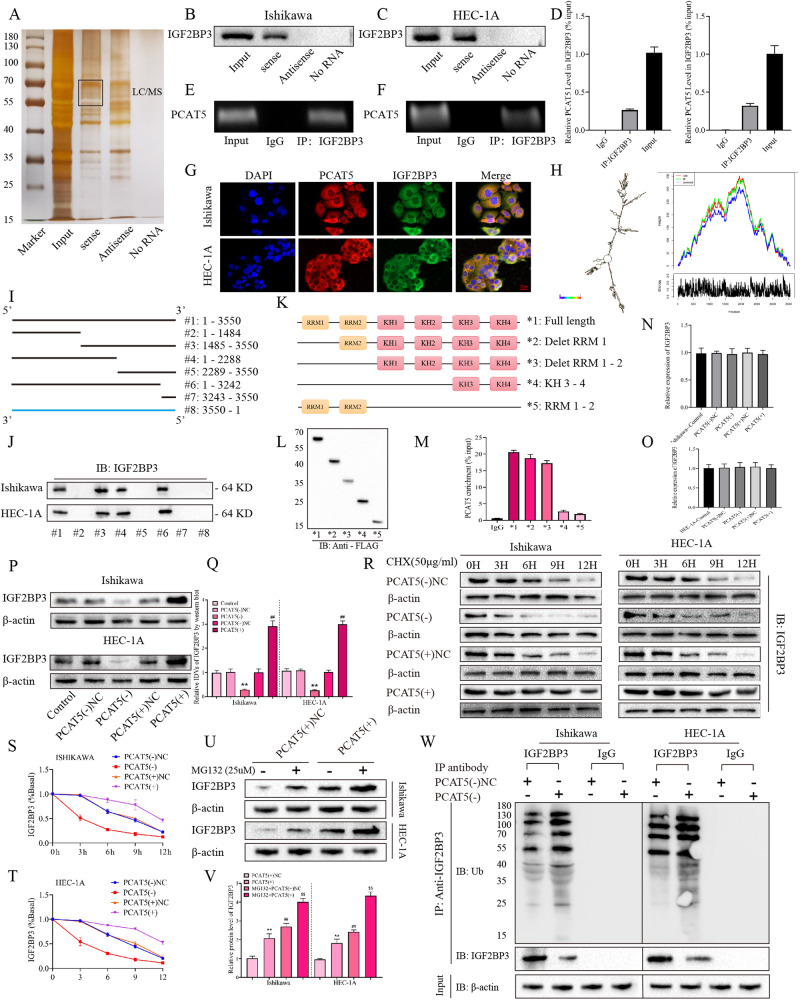
PCAT5 could directly bind to IGF2BP3 and inhibit ubiquitin/proteasome mediated the degradation of IGF2BP3. **A** The RNA-associated proteins were detected using SDS–PAGE and silver staining after RNA-pulldown. **B**, **C** In EC cells, IGF2BP3 was pulled down using the sense-PCAT5 RNA probe only (*n* = 3). **D**–**F** RIP-PCR assay was performed to investigate the binding of the IGF2BP3 antibody to PCAT5 in EC cells (*n* = 3). **G** FISH and IF double staining was performed to detected the binding effects between PCAT5 and IGF2BP3 in EC cells (*n* = 3) Scar bar = 25 μm. **H** The PCAT5 secondary structure was predicted on the website (http://www.lncipedia.org/). **I**, **J** Immunoblotting of IGF2BP3 in pull-down samples by full-length biotinylated-sense-PCAT5 (#1), truncated biotinylated. PCAT5 RNA motifs (#2- #7) or antisense-PCAT5 (#8). **K**–**M** Deletion mapping for the domains of IGF2BP3 that bind to PCAT5. RIP analysis for PCAT5 enrichment in cells transiently transfected with plasmids containing the indicated FLAG-tagged full-length or truncated constructs. **N**, **O** The effects of PCAT5 overexpression or silencing on the IGF2BP3 expression in EC cells was assessed by RT-qPCR (*n* = 3). **P**, **Q** The effects of PCAT5 overexpression or silencing on the IGF2BP3 expression in EC cells was assessed by Western blot (*n* = 3). **R**–**T** The effects of PCAT5 overexpression or silencing and CHX treatment on IGF2BP3 protein expression in were detected by Western blot (*n* = 3). **U**, **V** The effects of PCAT5 overexpression and MG132 treatment on IGF2BP3 protein were detected by Western blot (*n* = 3). **W** Cell lysates were immunoprecipitated with a control IgG or IGF2BP3 antibody and then immunoblotted with ubiquitin or IGF2BP3 antibody, with β-actin as controls (n = 3). Data are expressed as means ± standard deviation. *p < 0.05; **p < 0.01; ***p < 0.001.

Previous studies have found that lncRNAs expressed in the cytoplasm often play a role by acting as competitive endogenous RNA (ceRNA) or a scaffold for RBP [29]. In order to further study the specific mechanism of PCAT5 promoting EC progress. We performed an RNA-pulldown assay in HEK293T cells overexpressing PCAT5 using biotin-labeled d PCAT5 probes, then the differential bands were analyzed by silver staining, and the samples were analyzed by LC–MS. It was found that IGF2BP3 could be pulldown by the sense PCAT5 probe (Fig. 4A). Furthermore, the RIP and RNA-pulldown assays showed that PCAT5 could bind to IGF2BP3 in EC cell lines (Fig. 4B–F). In addition, the combination of PCAT5 and IGF2BP3 in the cytoplasm was confirmed by a FISH + IF double staining experiment (Fig. 4G).

Then, we analyzed the PCAT5 secondary structure (http://www.lncipedia.org/; Fig. 4H), and divided PCAT5 into fragments of different regions (#1–#8; Fig. 4I). Then pulldown experiments were carried out, respectively. Notably, it was found that only 1485-2288 nt fragments could be combined with IGF2BP3 (Fig. 4J). IGF2BP3 was made up of two RNA recognition motifs (RRMs) and four K homology domains (KHs) [30]. Therefore, we established five FLAG-labeled full-length or fragmented IGF2BP3 plasmids and transfected them into the HEK293T cell line and verified them by western blot (Fig. 4K, L). Further, through the RIP-qPCR experiment, it was found that PCAT5 was mainly enriched in the KH1–2 domain of IGF2BP3 (Fig. 4M). These results demonstrated the direct binding of PCAT5 to IGF2BP3. Then, we found that overexpression or knockdown of PCAT5 had no significant effect on the transcription level of IGF2BP3 in cells (Figs. 4N, O). However, overexpression of PCAT5 could significantly increase the protein expression of IGF2BP3, while knockdown of PCAT5 could significantly inhibit the expression of IGF2BP3 (Fig. 4P, Q). Thus, we hypothesized that PCAT5 might be involved in post-transcriptional regulation of IGF2BP3. Then, we added CHX, a protein synthesis inhibitor, to the cells and found that compared with the NC group, overexpression of PCAT5 could significantly prolong the half-life of IGF2BP3, while knockdown of PCAT5 could significantly shorten its half-life (Fig. 4R–T). Adding proteasome inhibitor-MG132 to the cells, they found that overexpression of PCAT5 could further increase the expression of IGF2BP3 (Fig. 4U, V). These results suggest that PCAT5 may inhibit the degradation of IGF2BP3 from entering the proteasome system. Finally, we carried out immune-co-precipitation experiments with IGF2BP3 antibody and immunoblotting with ubiquitin antibody and found that knockdown of PCAT5 could increase the ubiquitination level of IGF2BP3 (Fig. 4W).

### PCAT5 stabilizes IGF2BP3 by preventing MKRN2-mediated ubiquitination

**Fig. 5:**
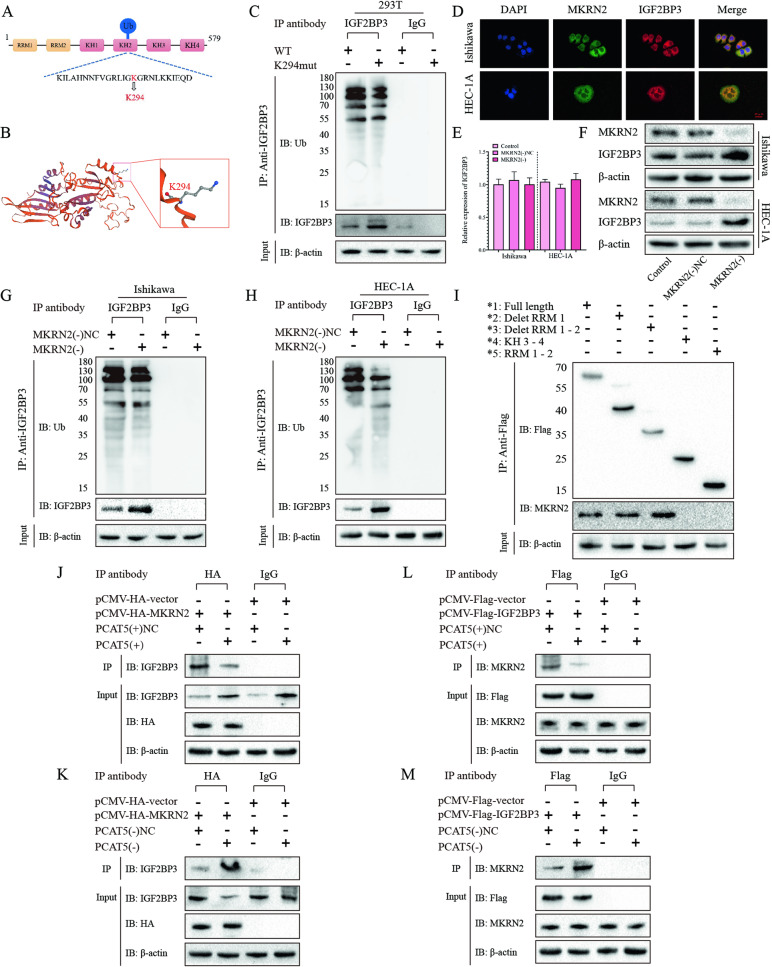
PCAT5 stabilizes IGF2BP3 by preventing MKRN2-mediated ubiquitination. **A**, **B** We predicted the structure of IGF2BP3 and visualize the K294 ubiquitination site on the website (https://swissmodel.expasy.org/). **C** We have mutation the K294 site of IGF2BP3 in HEK293T cells, then the Cell lysates were immunoprecipitated with a control IgG or IGF2BP3 antibody and then immunoblotted with ubiquitin or IGF2BP3 antibody, with β-actin as controls (*n* = 3). **D** IF double staining was performed to detected the binding effects between MKRN2 and IGF2BP3 in EC cells (*n* = 3) Scar bar = 25 μm. **E**, **F** The effects of MKRN2 silencing on the IGF2BP3 expression in EC cells was assessed by RT-qPCR and Western blot (*n* = 3). **G**, **H** The effects of MKRN2 silencing on the ubiquitination level of IGF2BP3 expression in EC cells was assessed by immunoprecipitated assays (*n* = 3). **I** Deletion mapping for the domains of IGF2BP3 that bind to MKRN2 with transiently transfected with plasmids containing the indicated FLAG-tagged full-length or truncated constructs; **J**–**M** Immunoprecipitation to detect the association between MKRN2 and IGF2BP3 after PCAT5 overexpression or knockdown. (*n* = 3). Data are expressed as means ± standard deviation. *p < 0.05; **p < 0.01; ***p < 0.001.

In order to further explore the molecular mechanisms of PCAT5 affecting the ubiquitination of IGF2BP3, we predicted the IGF2BP3 functional ubiquitination site through the website (https://www.phosphosite.org/), and discovered that K294 was an obvious ubiquitination site located on the KH2 domain of IGF2BP3 (Fig. 5A). In addition, we use the Swiss model website (https://swissmodel.expasy.org/) to predict the structure of IGF2BP3 and visualize the K294 ubiquitination site (Fig. 5B). Additionally, we mutated K294 in the HEK293T cell line, and then immunoprecipitation was performed with IGF2BP3 antibody and immunoblotting with ubiquitin antibody. This showed that mutated K294 significantly reduced the ubiquitination level of IGF2BP3, thus proving that K294 was a functional ubiquitination site of IGF2BP3 (Fig. 5C).

A recent report showed that MKRN2 is an E3 ligase that mediates the degradation of IGF2BP3 into the ubiquitin proteasome system [31, 32], which prompted us to explore whether PCAT5 has a role in regulating the interaction between MKRN2 and IGF2BP3 in EC cells. First, the co-location of IGF2BP3 and MKRN2 in the cytoplasm of EC cells was proved by an IF double staining experiment (Fig. 5D). Then, through RT-qPCR, we showed that knockdown of MKRN2 had no significant effect on the transcription level of IGF2BP3 (Fig. 5E), while knockdown of MKRN2 could significantly increase the protein expression level of IGF2BP3 (Fig. 5F). Further, the immunoprecipitation experiments showed that knockdown of MKRN2 could reduce the ubiquitination level of IGF2BP3 (Fig. 5G, H), which proved that MKRN2 induced the ubiquitination of IGF2BP3 in EC cells. In order to verify the binding region of MKRN2 and IGF2BP3, we established five full-length and fragment IGF2BP3 plasmids labeled with FLAG and transfected them into HEK293T cell lines. An IP experiment was performed with FLAG antibody, and immunoblotting with MKRN2 antibody, which proved that MKRN2 could be binding with IGF2BP3 KH1–2 domain binding (Fig. 5I). Finally, we used HA and FLAG to label MKRN2 and IGF2BP3 respectively, and carried out immunoprecipitation experiments. It was found that overexpression of PCAT5 could significantly reduce the binding of MKRN2 to IGF2BP3, while knockdown of PCAT5 could increase the binding between them (Fig. 5J–M). Taken together, these results suggest that PCAT5 could improve its stability by preventing MKRN2 mediated ubiquitin and proteasome-dependent degradation of IGF2BP3.

### PCAT5 promotes glycolysis, proliferation, migration, and invasion of EC cells by stabilizing IGF2BP3

**Fig. 6:**
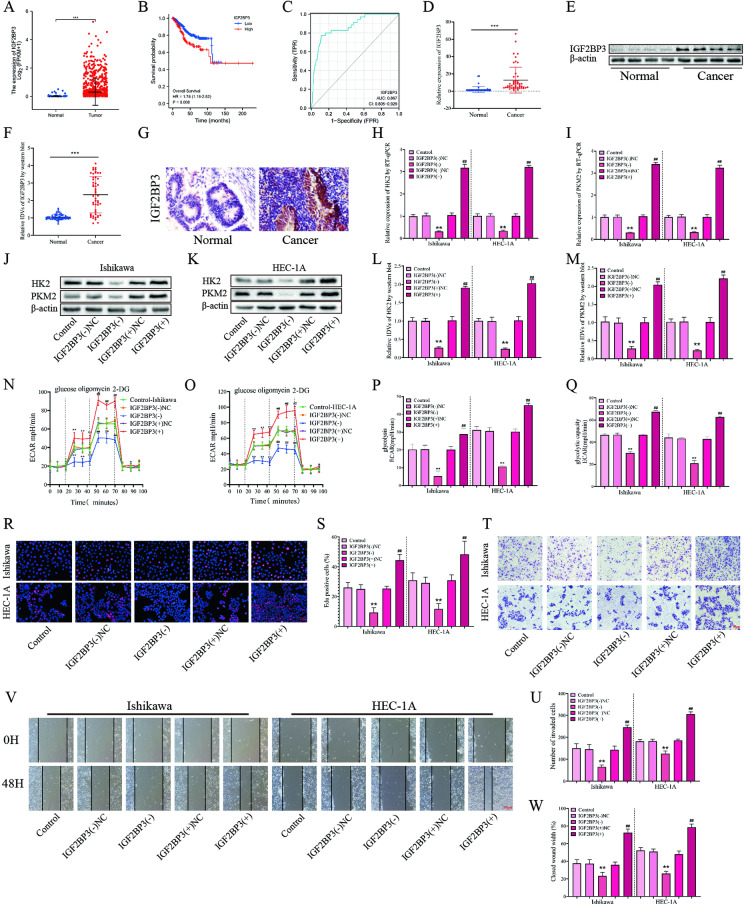
The expression of IGF2BP3 and its efects on glycolysis, proliferation migration and invasion in EC. **A** Expression of IGF2BP3 in TCGA cohort of endometrial tissues, tumor (*n* = 552), compared with normal endometrial tissue (*n* = 35). **B** Effect of IGF2BP3 expression level on EC patient survival time from TCGA database. **C** ROC of IGF2BP3 (TCGA cohort). **D** The *IGF2BP3* expression levels in the EC (*n* = 50) and control (*n* = 49) group were detected by RT-qPCR. **E**, **F** The protein expression levels of IGF2BP3 in the EC (*n* = 50) and control (*n* = 49) group were detected by western blot. **G** The expression of IGF2BP3 was detected via immunohistochemistry in EC (*n* = 50) and control (*n* = 49), Scar bar = 25 μm. **H**–**I** The effects of IGF2BP3 overexpression or silencing on the HK2 and PKM2 transcription in EC cells was assessed by RT-qPCR (*n* = 3). **J**–**M** The effects of IGF2BP3 overexpression or silencing on the HK2 and PKM2 expression in EC cells was assessed by western blot (*n* = 3). **N**, **Q** The effects of IGF2BP3 overexpression or silencing on the glycolysis ability in EC cells was assessed by ECAR (*n* = 3). **R**, **S** The effects of IGF2BP3 overexpression or silencing on the proliferation ability in EC cells was assessed by EdU assay (*n* = 3), Scar bar = 50 μm. **T**, **U** The effects of IGF2BP3 overexpression or silencing on the migration ability in EC cells was assessed by wound-healing assay (*n* = 3), Scar bar = 100 μm. **V**, **W** The effects of IGF2BP3 overexpression or silencing on the transwell ability in EC cells was assessed by transwell assay (*n* = 3), Scar bar = 50 μm. Data are expressed as means ± standard deviation. *p < 0.05; **p < 0.01; ***p < 0.001.

By Spearman rank correlation coefficient analysis, the expression level of PCAT5 and the protein expression level of IGF2BP3 in EC tissues in this study group, it is found that there is a positive correlation between them (Fig S2H). Then, TCGA further confirms our finding (Fig. 6A). High expression of IGF2BP3 was correlated with worse overall survival of EC patients (Fig. 6B). In addition, we found that IGF2BP3 has a high specificity and sensitivity in diagnosing EC (Fig. 6C). Further, RT-qPCR and western blot experiments showed that the transcription and expression of IGF2BP3 in the EC group was significantly higher than in the control group (Fig. 6D–F). In addition, immunohistochemistry showed that IGF2BP3 was mainly expressed in the cytoplasm of EC, and its expression was significantly higher than that of the control group (Fig. 6G, Table S9). Then, we analyzed the clinicopathological parameters of IGF2BP3 and EC patients and found that their expression was significantly correlated with clinicopathological features (Table S10). The expression of IGF2BP3 increased significantly as clinical stages progressed from I + II to III + IV. Compared with the highly differentiated group, the expression of IGF2BP3 was significantly increased in the moderately and poorly differentiated groups. Additionally, the high expression of IGF2BP3 was positively correlated with the degree of invasion, lymph node, and distant metastasis (Table S9). These results suggest that IGF2BP3 could significantly promote the occurrence and development of EC.

To investigate the effects of IGF2BP3 on glycolysis, proliferation, migration, and invasion of EC cells. We established Ishikawa and HEC-1A cell lines with stable overexpression or knockdown of IGF2BP3 using lentiviruses. Then, through RT-qPCR and western blot experiments, it was shown that knockdown of IGF2BP3 could inhibit the transcription and expression of HK2 and PKM2 (Fig. 6H–M). However, knocking down IGF2BP3 had no significant effect on the nascent RNA of HK2 and PKM2 (Fig S2I–J), but could significantly shorten the HK2 and PKM2 half-life of mRNA (Fig S2K–N). Further, the ECAR glucose consumption and lactic acid concentration rates showed that knocking down IGF2BP3 could significantly inhibit the glycolysis ability of EC cells (Fig. 6N–Q, Fig S2O–P). Finally, through the EdU, wound-healing, and transwell assays, we proved that knockdown of IGF2BP3 could inhibit the proliferation, migration, and invasion ability of EC cells, while overexpression of IGF2BP3 could produce the opposite effect (Fig. 6R–W).

To investigate whether IGF2BP3 affects the mechanism of PCAT5’s ability to promote EC glycolysis, proliferation, migration, and invasion. We transfected IGF2BP3 shRNA or NC into the EC cell lines overexpressing PCAT5. Then, through the RT-qPCR and Western blot experiments, it was shown that knocking down IGF2BP3 could offset the transcription and expression activation of HK2 and PKM2 caused by overexpression of PCAT5 (Fig S3A–E). In addition, through the ECAR, glucose consumption, and lactate concentration rate experiments, it was found that knockdown of IGF2BP3 could counteract the ability of overexpressing PCAT5 to promote glycolysis of EC cells (Fig S3F–I, Fig S2Q–R). Finally, Edu wound-healing and transwell experiments revealed that knocking down IGF2BP3 might counteract the effect of PCAT5 overexpression on EC cell migration and invasion (Fig S3J–O).

### Tumor xenografts in nude mice

**Fig. 7:**
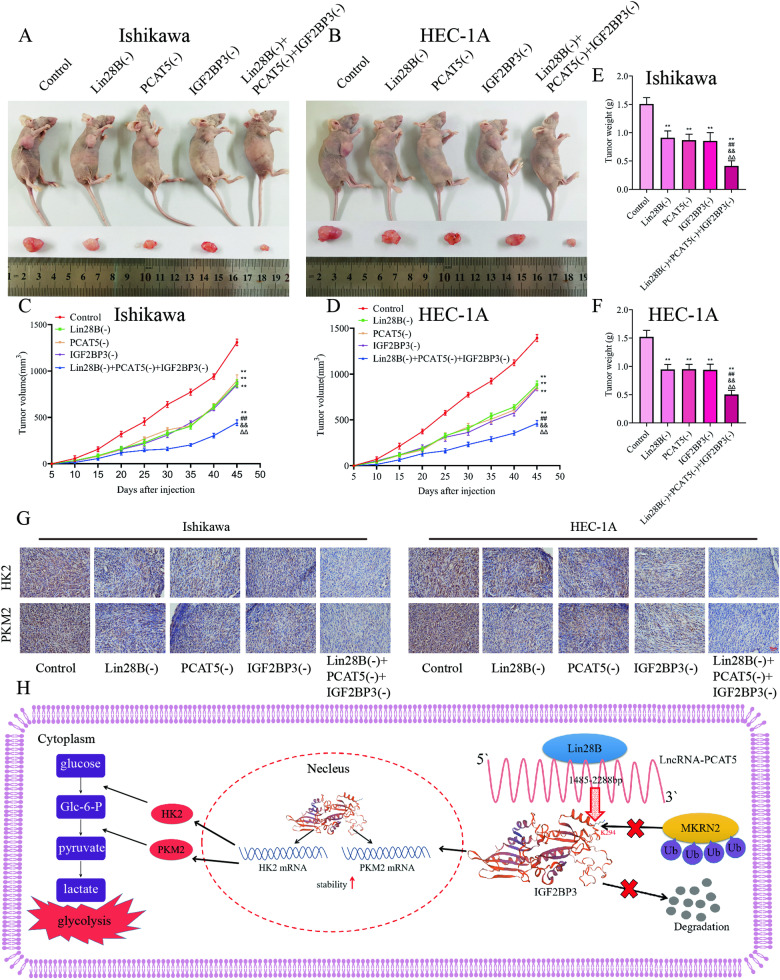
Simultaneous knockdown of LIN28B PCAT5 and IGF2BP3 can inhibit tumor growth in vivo. **A**, **B** Subcutaneously xenografted nude mice injected with different treated cells are shown (*n* = 3, each group). **C**, **D** Tumor growth curves are shown. **E**, **F** Tumor average weight are shown; Tumor size was recorded every 5 days, and tumors were extracted at 45 days after injection. **G** Immunohistochemical staining on the xenograft tumor from nude mice was performed to detected the effects of HK2 and PKM2 expression by knockdown of LIN28B, PCAT5 or IGF2BP3. **H** Mechanism diagram of LIN28B / PCAT5 / IGF2BP3 axis as a potential glycolytic metabolic regulator in EC. Data are expressed as means ± standard deviation. *p < 0.05; **p < 0.01; ***p < 0.001.

In order to confirm the role of LIN28B, PCAT5, and IGF2BP3 in tumor growth in vivo, we subcutaneously injected Ishikawa or HEC-1A cells with knockdown of LIN28B, PCAT5 and IGF2BP3 respectively, or a combined knockdown of LIN28B + PCAT5 + IGF2BP3 to construct a xenograft tumor model in nude mice. The results showed that, when compared to the control group, knockdown of LIN28B, PCAT5, or IGF2BP3 alone could significantly reduce the average volume of xenografts in nude mice, while the combined knockdown of LIN28B + PCAT5 + IGF2BP3 group had the smallest volume of xenografts (Fig. 7A–D). Similarly, compared to the control group, knockdown of LIN28B, PCAT5, or IGF2BP3 alone could significantly reduce the average weight of xenografts in nude mice, with the combined knockdown of LIN28B + PCAT5 + IGF2BP3 group having the lightest weight (Fig. 7E–F).

In addition, we performed immunohistochemical staining on the xenograft tumor from nude mice and found that compared with the control group, knockdown of LIN28B, PCAT5, or IGF2BP3 alone could significantly reduce the expression of HK2 and PKM2 in the nude xenograft tumor, while the expression of HK2 and PKM2 in the combined knockdown of the LIN28B + PCAT5 + IGF2BP3 group was the lowest (Fig. 7G).

To further demonstrate in vivo that the “LIN28B/ PCAT5/ IGF2BP3 axis promotes aerobic glycolysis, we performed rescue experiments in vivo. Compared with the control group, Lin28B knockdown reduce the average volume of xenografts in nude mice, while overexpression of PCAT5 can save the tumor growth inhibition of Lin28B knockdown (Fig S4A–D). moreover, compared with the control group, PCAT5 knockdown significantly reduce the average volume of xenografts in nude mice, and overexpression of IGF2BP3 could save tumor growth inhibition of PCAT5 knockdown (Fig S4A–D). In addition, immunohistochemical staining was performed on the transplanted tumor of nude mice, and it was found that the expression of HK2 and PKM2 in the transplanted tumor of nude mice could be significantly reduced by knockdown of Lin28B compared with the control group, while simultaneously overexpression of PCAT5 could save the decrease of HK2 and PKM2 (Fig S4E). Moreover, compared with the control group, the expression of HK2 and PKM2 could be significantly reduced by lowering PCAT5, while the overexpression of IGF2BP3 could save the decrease of HK2 and PKM2 (Fig S4E).

Finally, we summarized the mechanism diagram of LIN28B / PCAT5 / IGF2BP3 axis as a potential glycolytic metabolic regulator in EC (Fig. 7H).

## Discussion

The prognosis of EC mainly depends on the stage of diagnosis and histology. Most EC patients in stages I and II have a favorable prognosis, whereas those in stages III or IV have a poor prognosis [33]. With the progress of EC molecular research, the genomic changes of EC have been revealed, and valuable insights have been provided into the pathogenesis of the disease. Early detection of endometrial cancer or precursors using molecular diagnosis may be especially useful in guiding personalized EC therapy [34]. In this study, we found for the first time that LIN28B expression was significantly correlated with clinicopathological features. The expression of LIN28B increased with clinical stage progression and was significantly elevated in patients with lymph node metastasis. Additionally, we found for the first time that the expression of PCAT5 increased with the progress of clinical stage, and the expression of PCAT5 was significantly higher in the medium and low differentiation groups than in the highly differentiated group, and was positively correlated with the degree of invasion, and high expression of PCAT5 was significantly correlated with lymph nodes and distant metastasis. It is suggested that LIN28B and PCAT5 may have the potential as biomarkers of EC. Similar to previous studies [20, 21], IGF2BP3 was also closely related to clinicopathological parameters in our study.

The reprogramming of glucose metabolism plays an important role in promoting the malignant characteristics of tumors. Studies have found that LIN28B plays a basic role in maintaining the glycolytic metabolism of tumor stem cells. Knocking down LIN28B in vivo and in vitro can inhibit extracellular acidification rate, glucose uptake, and lactate production [12]. Recent studies have found that LIN28B promotes aerobic glycolysis and reduces oxidative phosphorylation in osteosarcoma cells. In OS13 cells, knocking down LIN28B can reduce tumor occurrence, reduce chemotherapy resistance, and reverse oxidative phosphorylation [13]. In EC, LIN28B was identified as the direct target of miR-152-3p, and a negative correlation was found between LIN28B and miR-152-3p. In addition, overexpression of miR-152-3p can inhibit EC progression by directly targeting LIN28B [11]. In this study, we found for the first time that LIN28B can promote the transcription and expression of key glycolysis enzymes HK2 and PKM2 in EC cells, thereby promoting the capacity of aerobic glycolysis and promoting malignant biological behavior. Through lncRNA microarray, RIP, and RNA-pulldown experiments, we determined that LIN28B could bind to PCAT5 in EC cells. A large number of studies have shown that RBP is a key regulator of transcription and post-transcriptional processing [35]. RBP can regulate RNA splicing, polyadenylation, mRNA stability, mRNA localization, and mRNA translation by interacting with coding and non-coding RNA and other proteins [36]. Mechanistically, we found that LIN28B can stabilize PCAT5, thus prolonging the half-life of PCAT5.

In addition, many evidences indicates that lncRNAs play an important functional role in regulating the transcription and translation of metabolically related genes, and can act as bait, scaffolders, or ceRNAs, ultimately leading to tumor metabolic reprogramming [15]. In EC, lncRNA-DLEU2 can act as a ceRNA of miR-455 to induce HK2 expression and promote aerobic glycolysis by regulating the EZH2/miR-181a pathway [4]. Another study found that SNHG16 could up-regulate the target gene HK2 by sponging miR-490-3p, thereby promoting the proliferation and glycolysis of EC [37]. In this study, we discovered that PCAT5 can promote the transcription and expression of HK2 and PKM2 in EC cells, thus promoting the capacity of aerobic glycolysis and promoting malignant biological behavior. Many studies have shown that lncRNAs can regulate the expression of protein-coding genes at epigenetic and post-transcriptional levels [38]. We identified that PCAT5 could directly bind to IGF2BP3 in cells by RNA-pulldown, LC–MS, and RIP experiments. PCAT5 was discovered to be involved in post-transcriptional regulation of IGF2BP3 in terms of mechanism.

Multiple studies have found that IGF2BP3 is post-transcriptionally regulated in tumors [23, 31, 39]. In gliomas, circNEIL3 could promote tumorigenesis by blocking HECD4-mediated IGF2BP3 ubiquitination [23]. In colon cancer, USP11 could bind IGF2BP3 and block its ubiquitination degradation, which then promotes tumor proliferation and metastasis [39]. MKRN2, an E3 ligase, was found to be involved in the ubiquitination of IGF2BP3 in neuroblastoma. MKRN2 silencing promotes tumor proliferation and migration by decreasing ubiquitination of IGF2BP3 [31]. MKRN2 was associated with conserved RING finger domains that control a variety of molecules through the ubiquitin-proteasome system, including p53, P21, FADD, PTEN, P65, Nptx1, GLK, and some viral or bacterial proteins [32]. In this study, we found for the first time that MKRN2 can bind to IGF2BP3 in EC cells and mediate its ubiquitination. Mechanistically, we found that PCAT5 binds to the KH1–2 domain of IGF2BP3, preventing MKRN2 from binding to the K294 ubiquitination site in the KH2 domain, thus stabilizing IGF2BP3 and prolonging its half-life. However, the expression of MKRN2 in EC has not been involved in this study, which needs to be further explored. IGF2BP3, as a member of RBP, has been known to stabilize mRNA of downstream target genes [40]. In this study, we demonstrated for the first time that IGF2BP3 can stabilize HK2 and PKM2 mRNA in EC, thereby increasing the level of aerobic glycolysis and promoting the ability of proliferation, migration, and invasion.

Finally, our in vivo study showed that knockdown of LIN28B, PCAT5, and IGF2BP3 could inhibit tumor growth in xenografted tumors, and mice injected with the combination of these three agents showed minimal xenografted tumor volume and weight. In addition, immunohistochemical experiments demonstrated that knockdown of LIN28B, PCAT5, and IGF2BP3 could inhibit HK2 and PKM2 expression in vivo tumors, further demonstrating the ability of the LIN28B/ PCAT5/ IGF2BP3 axis to promote aerobic glycolysis. These results suggest that knockdown of LIN28B, PCAT5, and IGF2BP3 has potential clinical value.

## Conclusion

In conclusion, Firstly, the potential of LIN28B, PCAT5, and IGF2BP3 as biomarkers of EC was established in this study. Secondly, in vivo experiments proved that knockdown of the LIN28B/ PCAT5/ IGF2BP3 axis has potential clinical value in the treatment of EC. Finally, it is found that LIN28B binds and stabilizes PCAT5. Then PCAT5 binds to the KH1–2 domain of IGF2BP3, preventing MKRN2 from binding to the K294 KH2 domain and thus stabilizing IGF2BP3 and prolonging its half-life. IGF2BP3 stabilizes the mRNA of HK2 and PKM2 and promotes aerobic glycolysis and malignant biological behavior of EC.

### Supplementary information

Original western blot band

Supplement table

Supplement Figure

aj-checklist

## Data Availability

The datasets used and/or analysed during the current study are available from the corresponding author on reasonable request.
