# Supplementary material for: LIN28B induced PCAT5 promotes endometrial cancer progression and glycolysis via IGF2BP3 deubiquitination
Source: Cell Death Dis. 2024 Apr 2;15(4):242. doi: 10.1038/s41419-024-06564-2 (PMC10987620; doi:10.1038/s41419-024-06564-2)
Supplement: Supplementary file 1 — Original western blot band [file 41419_2024_6564_MOESM1_ESM.pdf]

Figure 1F

LIN28B β-actin

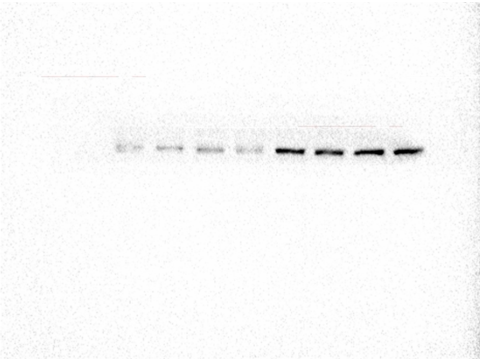

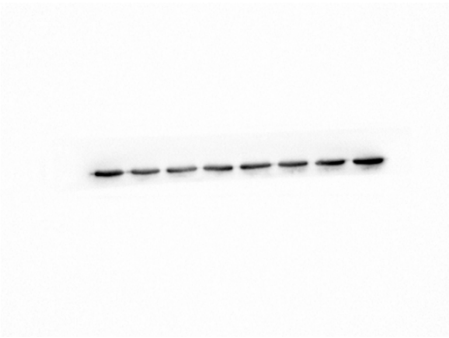

Figure 1J

Ishikawa

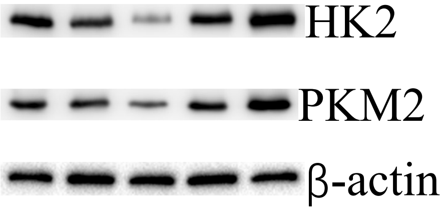

HK2

PKM2

β-actin

HEC-1A

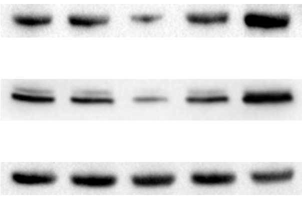

HK2

PKM2

β-actin

Figure 2L

Ishikawa

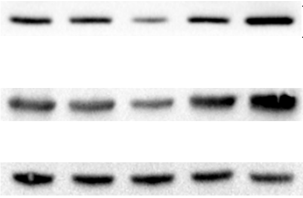

HK2

PKM2

β-actin

HEC-1A

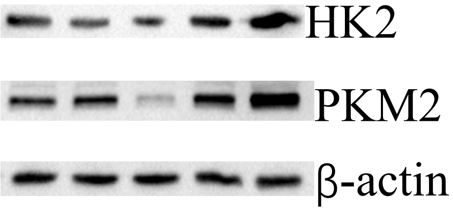

HK2

PKM2

β-actin

Figure 3B

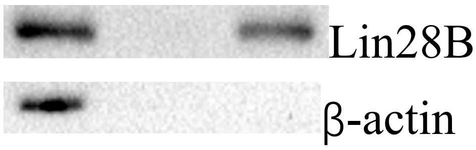

Lin28B

β-actin

Figure 3H

Ishikawa

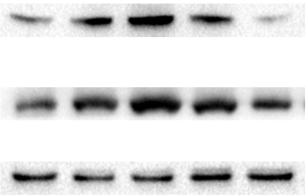

HK2

PKM2

β-actin

HEC-1A

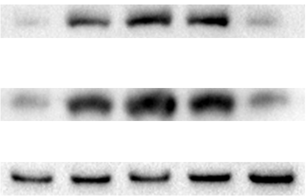

HK2

PKM2

β-actin

Figure 4B

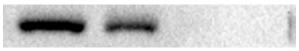

IGF2BP3

Figure 4C

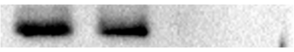

Figure 4E

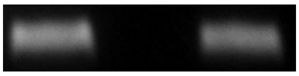

Figure 4F

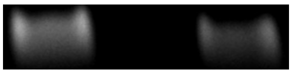

Figure 4J

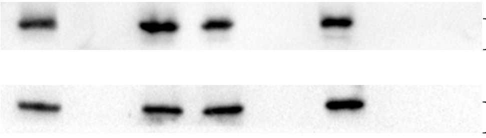

Figure 4L

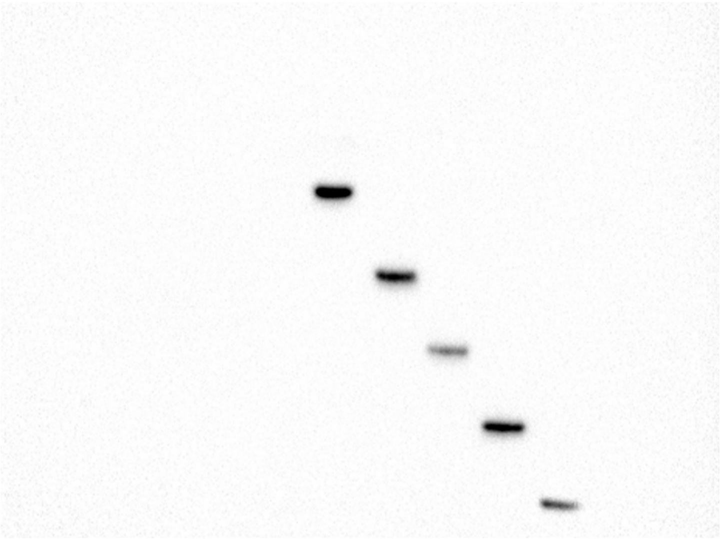

Figure 4P

Ishikawa

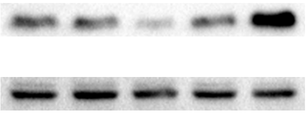

HEC-1A

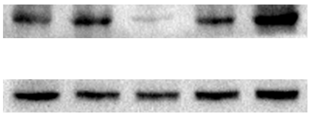

Figure 4R

Ishikawa

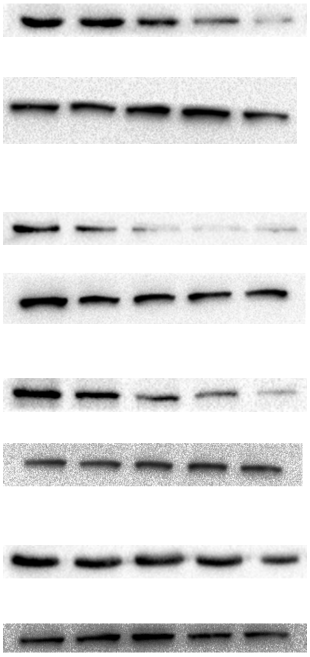

HEC-1A

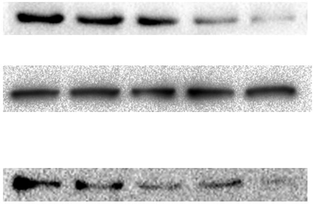

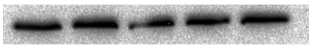

β-actin

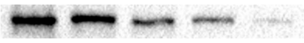

PCAT5(+)NC

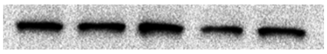

β-actin

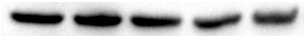

PCAT5(+)

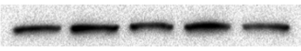

β-actin

Figure 4U

Ishikawa

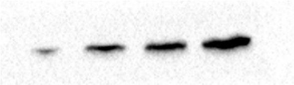

IGF2BP3

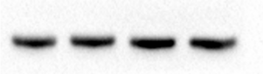

β-actin

HEC-1A

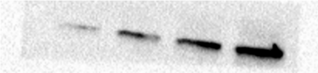

IGF2BP3

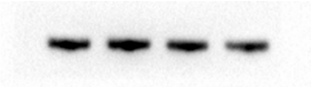

β-actin

Figure 4W

Ishikawa

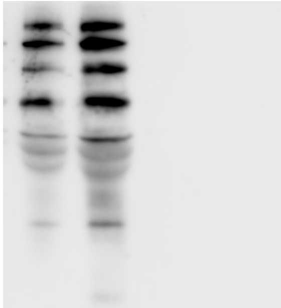

Ub

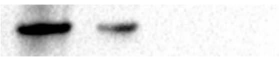

IGF2BP3

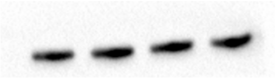

β-actin

HEC-1A

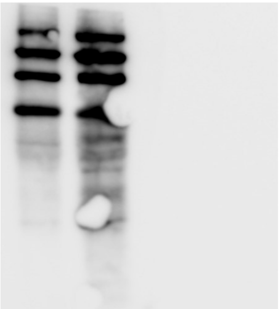

Ub

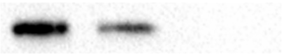

IGF2BP3

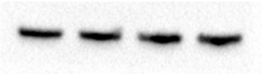

β-actin

Figure 5C

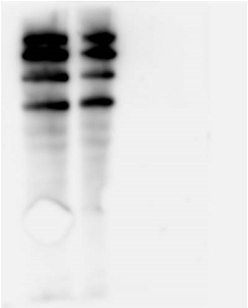

Ub

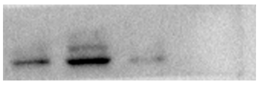

IGF2BP3

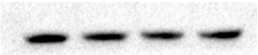

β-actin

Figure 5F

Ishikawa

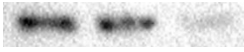

MKN2

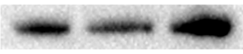
IGF2BP3

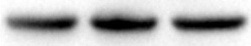
β-actin

HEC-1A

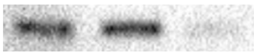
MKN2

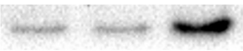
IGF2BP3

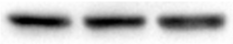
β-actin

Figure 5G

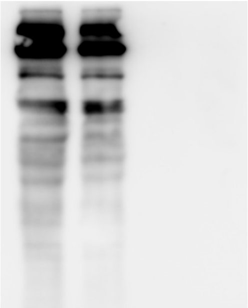
Ub

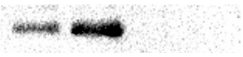
IGF2BP3

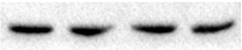
β-actin

Figure 5H

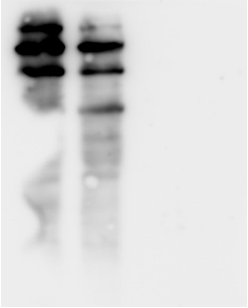
Ub

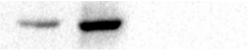
IGF2BP3

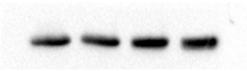
β-actin

Figure 5I

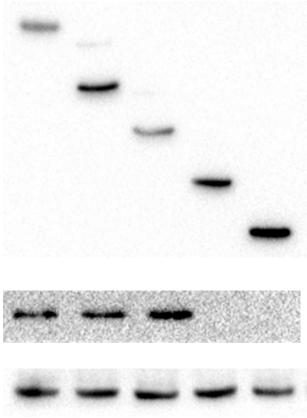

Figure 5J

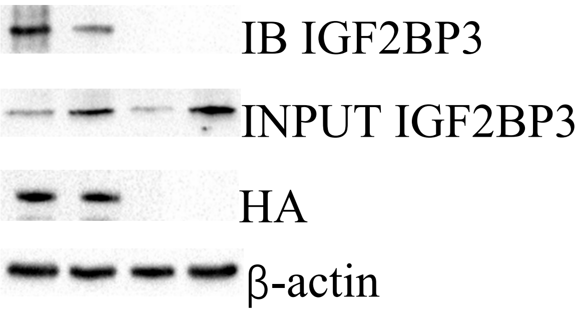

Figure 5K

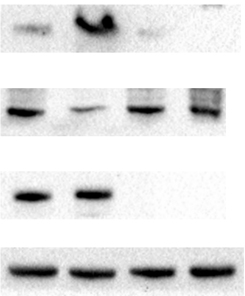

Figure 5L

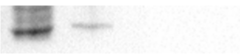

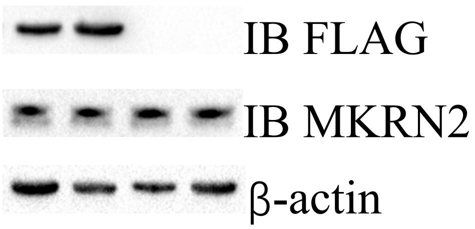

Figure 5M

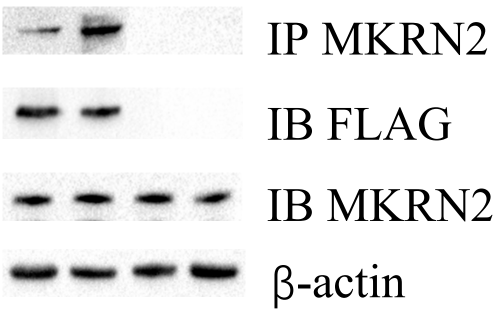

Figure 6E

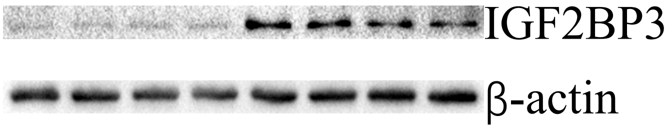

Figure 6J

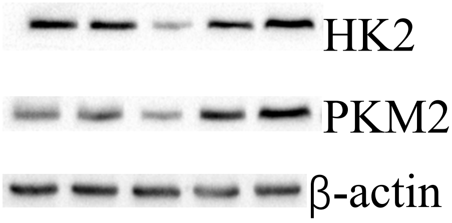

Figure 6K

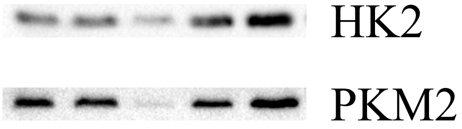

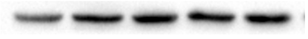 β-actin

Figure S3C

Ishikawa

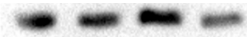 HK2

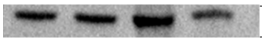 PKM2

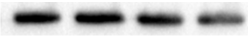 β-actin

HEC-1A

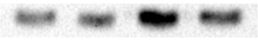 HK2

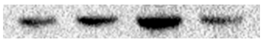 PKM2

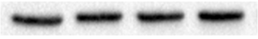 β-actin
