# Supplementary material for: LIN28B induced PCAT5 promotes endometrial cancer progression and glycolysis via IGF2BP3 deubiquitination
Source: Cell Death Dis. 2024 Apr 2;15(4):242. doi: 10.1038/s41419-024-06564-2 (PMC10987620; doi:10.1038/s41419-024-06564-2)
Supplement: Supplementary file 2 — Supplement table [file 41419_2024_6564_MOESM2_ESM.docx]

| Table S1 | |
| --- | --- |
| Sequences for the primers | |
| Primer names | sequence (5’→3’) |
| Lin28B-F | 5'-CAACTGTGGTGGCCTTGATC-3' |
| Lin28B-R | 5'-TGCTTCCTGTCTTCCCTGAG-3' |
| PCAT5-F | 5'-TCTAAGCACTGCAACTGGGA-3' |
| PCAT5-R | 5'-TGGCAAATGTTCAGCAGCAT-3' |
| DLEU2-F | 5'-ATGGTCCCTGTCAGCAAAGA-3' |
| DLEU2-R | 5'-GCGGCAATGGTGGTATCAAA-3' |
| H19-F | 5'-TCCTGAACACCTTAGGCTGG-3' |
| H19-R | 5'-TTCACCTTCCAGAGCCGATT-3' |
| NBAT1-F | 5'-TAAGTGGCCCCTCTGATTGG-3' |
| NBAT1-R | 5'-CATCACAGACAGGGCCCTAT-3' |
| IGF2BP3-F | 5'-TGGAAAAGGAGGCAAAACGG-3' |
| IGF2BP3-R | 5'-CTTCTGTTGTTGGTGCTGCT-3' |
| HK2-F | 5'-GACCAACTTCCGTGTGCTTT-3' |
| HK2-R | 5'-TCCATGAAGTTAGCCAGGCA-3' |
| PKM2-F | 5'-ATGGCTGACACATTCCTGGA-3' |
| PKM2-R | 5'-AGAAGTTCAGACGAGCCACA-3' |
| β-actin-F: | 5'-CATCCGCAAAGACCTGTACG-3' |
| β-actin-R: | 5'-CCTGCTTGCTGATCCACATC-3' |
|  |  |
| The short hairpin RNAs against Lin28B, PCAT5, IGF2BP3 and MKRN2 sites | |
| Gene | sequence (5’→3’) |
| Lin28B #1 | 5'-GGATATTCCAGTCGATGTATT-3' |
| Lin28B #2 | 5'-GGAGATAGATGCTACAACTGT-3' |
| Lin28B #3 | 5'-GCCATTACTGTCAGAGCATCA-3' |
| PCAT5 #1 | 5'-GCAAGATCAGGGAAGCAAACC-3' |
| PCAT5 #2 | 5'-GGATGTTACAACAGAGCTTGC-3' |
| PCAT5 #3 | 5'-GCAGCGGTGGGAACAATTTAA-3' |
| IGF2BP3 #1 | 5'-GCAAAGGATTCGGAAACTTCA-3' |
| IGF2BP3 #2 | 5'-GGATTCGGAAACTTCAGATAC-3' |
| IGF2BP3 #3 | 5'-GGTTCCCACCCAATTTGTTGG-3' |
| MKRN2 #1 | 5'-GCAATCACACGTACTGTTTGT-3' |
| MKRN2 #2 | 5'-GCGGTGTGCCAAACAGTTTGA-3' |
| MKRN2 #3 | 5'-GCCGTGTGATATCAGAGTTTG-3' |
|  |  |
| FISH probe | |
| Gene | sequence (5’→3’) |
| PCAT5 | 5'-GTAGCTGGGATTACAGGTGCCTTCTAGCACGCCCAGC-3' |
|  |  |
| RNA-puldown probes | |
| Gene | sequence (5’→3’) |
| #1-PCAT5(1-3550nt)-Pulldown-sense-F: | 5' CACGCCACTGCACTCTAGCCTGGGCAACAAG 3' |
| #1-PCAT5(1-3550nt)-Pulldown-sense-R: | 5' CCTGATCTTGCTGCAGCCTCAGGAG 3' |
| #2-PCAT5(1-1484nt) Pulldown-F: | 5' CACGCCACTGCACTCTAGCCTGGGCAACAAG 3' |
| #2-PCAT5(1-1484nt) Pulldown-R: | 5' GAATTAAATTGTTCCCACCGCTGCTCTGCTCCTTCA 3' |
| #3-PCAT5(1485-3550nt) Pulldown-F: | 5' AAGTTGGGTAATTGATGTCTTCATGCTTCCACAAAGGGC 3' |
| #3-PCAT5(1485-3550nt) Pulldown-R: | 5' GAATTAAATTGTTCCCACCGCTGCTCTGCTCCTTCA 3' |
| #4-PCAT5(1-2288nt) Pulldown-F: | 5' CACGCCACTGCACTCTAGCCTGGGCAACAAG 3' |
| #4-PCAT5(1-2288nt) Pulldown-R: | 5' GAATTAAATTGTTCCCACCGCTGCTCTGCTCCTTCA 3' |
| #5-PCAT5(2289-3550nt) Pulldown-F: | 5' AAGTTGGGTAATTGATGTCTTCATGCTTCCACAAAGGGC 3' |
| #5-PCAT5(2289-3550nt) Pulldown-R: | 5' GAATTAAATTGTTCCCACCGCTGCTCTGCTCCTTCA 3' |
| #6-PCAT5(1-3242nt) Pulldown-F: | 5' CACGCCACTGCACTCTAGCCTGGGCAACAAG 3' |
| #6-PCAT5(1-3242nt) Pulldown-R: | 5' GAATTAAATTGTTCCCACCGCTGCTCTGCTCCTTCA 3' |
| #7-PCAT5(3243-3550nt) Pulldown-F: | 5' ATTTTCAAGGCTATGCAATTTAATGATACTGACAAACCCC 3' |
| #7-PCAT5(3243-3550nt) Pulldown-R: | 5' GAATTAAATTGTTCCCACCGCTGCTCTGCTCCTTCA 3' |
| #8-PCAT5(1-3550nt)-Pulldown-antisense-F： | 5' CACGCCACTGCACTCTAGCCTGGGCAACAAG 3' |
| #8-PCAT5(1-3550nt)-Pulldown-antisense-R： | 5' GAATTAAATTGTTCCCACCGCTGCTCTGCTCCTTCA 3' |

| Table S2 | | | | | |
| --- | --- | --- | --- | --- | --- |
| Antibodies for Immunoblotting, IHC, IF, RIP and IP | | | | | |
| Protein Name | Company | Catalog Number | Dilutions in WB | Dilutions in IHC/IF | Dilutions in RIP/IP |
| Lin28B | Proteintech | Cat No : 16178-1-AP | 1:1000 | 1:100 | 5μg |
| IGF2BP3 #1 | Abcam | ab177477 | 1:2000 | 1:100 | 5μg |
| IGF2BP3 #2 | Proteintech | Cat No. 66526-1-Ig | N/A | 1:50 | N/A |
| MKRN2 | Proteintech | Cat No. 12238-1-AP | 1:1000 | 1:50 | N/A |
| HA | Cell Signaling Technology | #3724 | 1:5000 | N/A | 5μg |
| Flag | Cell Signaling Technology | #8146 | 1:5000 | N/A | 5μg |
| HK2 | Proteintech | Cat No. 66974-1-Ig | 1:5000 | 1:100 | N/A |
| PKM2 | Proteintech | Cat No. 15822-1-AP | 1:1000 | 1:100 | N/A |
| Ubiquitin | Proteintech | Cat No. 10201-2-AP | 1:1000 | N/A | N/A |
|  |  |  |  |  |  |
| Secondary antibodies for Immunoblotting and IF | | | | |  |
| Company | Name | Catalog Number | Dilutions in WB | Dilutions in IF |  |
| Proteintech | Horseradish peroxidase conjugated secondary antibodies: goat anti-mouse | Cat No. SA00001-1 | 1:5000 | N/A |  |
| Proteintech | Horseradish peroxidase conjugated secondary antibodies: goat anti-rabbit | Cat No. SA00001-2 | 1:5000 | N/A |  |
| Abcam | Alexa Fluor^TM^ 488 | Ab150077 | N/A | 1:500 |  |
| Abcam | Alexa Fluor^TM^ 594 | Ab150116 | N/A | 1:500 |  |

| Table S3 | | | | | | | |
| --- | --- | --- | --- | --- | --- | --- | --- |
| lncRNA gene expression profiles as obtained from samples in sh-NC and sh-Lin28B groups as indicated | | | | | | | |
| Ishikawa-Lin28B(-)NC | | | | Ishikawa-Lin28B(-) | | | |
| gene_name | gene_id | log2FoldChange | padj | gene_name | gene_id | log2FoldChange | padj |
| IGF2-AS | ENSG00000099869 | 0.815632 | 0.00351 | IGF2-AS | ENSG00000099869 | -0.771451 | 0.015882 |
| H19 | ENSG00000130600 | 1.771516 | 0.00122 | H19 | ENSG00000130600 | -0.952136 | 0.003215 |
| NBAT1 | ENSG00000260455 | 1.210045 | 0.00214 | NBAT1 | ENSG00000260455 | -0.899116 | 0.017076 |
| DLEU2 | ENSG00000231607 | 1.355161 | 0.00016 | DLEU2 | ENSG00000231607 | -1.515144 | 0.000332 |
| MEG9 | ENSG00000223403 | 0.612321 | 0.00348 | MEG9 | ENSG00000223403 | -0.551610 | 0.000400 |
| PCAT5 | ENSG00000280719 | 2.100517 | 0.00016 | PCAT5 | ENSG00000280719 | -2.320014 | 0.000190 |
| LINC01606 | ENSG00000253301 | 0.541168 | 0.00232 | LINC01606 | ENSG00000253301 | -0.669119 | 0.013993 |
| PICSAR | ENSG00000275874 | 0.479915 | 0.00512 | PICSAR | ENSG00000275874 | -0.441211 | 0.014555 |
| GPR1-AS | ENSG00000279220 | 0.511237 | 0.00466 | GPR1-AS | ENSG00000279220 | -0.581588 | 0.018132 |
| PVT1 | ENSG00000249859 | 1.200127 | 0.00288 | PVT1 | ENSG00000249859 | -1.335114 | 0.000252 |
| ZNF295-AS1 | ENSG00000237232 | -1.110056 | 0.00581 | ZNF295-AS1 | ENSG00000237232 | 1.544001 | 0.013604 |
| LINC02231 | ENSG00000248995 | -0.451221 | 0.01211 | LINC02231 | ENSG00000248995 | 0.515001 | 0.012441 |
| PCAT19 | ENSG00000267107 | -0.661171 | 0.02113 | PCAT19 | ENSG00000267107 | 0.778991 | 0.002335 |
| HPAT5 | ENSG00000280707 | -0.789911 | 0.00522 | HPAT5 | ENSG00000280707 | 0.666998 | 0.001587 |
| LINC02166 | ENSG00000260259 | -1.332125 | 0.00345 | LINC02166 | ENSG00000260259 | 1.112166 | 0.005554 |
| CASC23 | ENSG00000255420 | -0.988187 | 0.01557 | CASC23 | ENSG00000255420 | 0.996663 | 0.004211 |
| LINC01503 | ENSG00000233901 | -1.588118 | 0.00223 | LINC01503 | ENSG00000233901 | 2.180220 | 0.003352 |
| PLUT | ENSG00000247381 | -0.299954 | 0.02215 | PLUT | ENSG00000247381 | 0.333214 | 0.002147 |
| LINC01366 | ENSG00000235172 | -0.778815 | 0.00322 | LINC01366 | ENSG00000235172 | 0.898911 | 0.001121 |
| ARLNC1 | ENSG00000260896 | -1.221336 | 0.03322 | ARLNC1 | ENSG00000260896 | 1.677156 | 0.004895 |
| HEC-1A-Lin28B(-)NC | | | | HEC-1A-Lin28B(-) | | | |
| gene_name | gene_id | log2FoldChange | padj | gene_name | gene_id | log2FoldChange | padj |
| IGF2-AS | ENSG00000099869 | 0.912216 | 0.00215 | IGF2-AS | ENSG00000099869 | -0.661156 | 0.002217 |
| H19 | ENSG00000130600 | 1.599914 | 0.01555 | H19 | ENSG00000130600 | -1.211166 | 0.003329 |
| NBAT1 | ENSG00000260455 | 1.355415 | 0.03221 | NBAT1 | ENSG00000260455 | -0.871919 | 0.002219 |
| DLEU2 | ENSG00000231607 | 1.588819 | 0.00267 | DLEU2 | ENSG00000231607 | -1.771191 | 0.006161 |
| MEG9 | ENSG00000223403 | 0.455512 | 0.00218 | MEG9 | ENSG00000223403 | -0.446191 | 0.004415 |
| PCAT5 | ENSG00000280719 | 2.589916 | 0.00234 | PCAT5 | ENSG00000280719 | -2.441116 | 0.000116 |
| LINC01606 | ENSG00000253301 | 0.663217 | 0.001 | LINC01606 | ENSG00000253301 | -0.766161 | 0.002118 |
| PICSAR | ENSG00000275874 | 0.744159 | 0.00222 | PICSAR | ENSG00000275874 | -0.389919 | 0.001447 |
| GPR1-AS | ENSG00000279220 | 0.422219 | 0.00022 | GPR1-AS | ENSG00000279220 | -0.511159 | 0.011147 |
| PVT1 | ENSG00000249859 | 1.369915 | 0.00021 | PVT1 | ENSG00000249859 | -1.455891 | 0.002691 |
| ZNF295-AS1 | ENSG00000237232 | -1.288818 | 0.00333 | ZNF295-AS1 | ENSG00000237232 | 1.611187 | 0.002117 |
| LINC02231 | ENSG00000248995 | -0.661158 | 0.00115 | LINC02231 | ENSG00000248995 | 0.499191 | 0.021161 |
| PCAT19 | ENSG00000267107 | -0.866619 | 0.01115 | PCAT19 | ENSG00000267107 | 0.811099 | 0.007449 |
| HPAT5 | ENSG00000280707 | -1.100018 | 0.00272 | HPAT5 | ENSG00000280707 | 0.766198 | 0.005889 |
| LINC02166 | ENSG00000260259 | -1.544191 | 0.01134 | LINC02166 | ENSG00000260259 | 1.344491 | 0.009879 |
| CASC23 | ENSG00000255420 | -0.881156 | 0.0037 | CASC23 | ENSG00000255420 | 1.022180 | 0.029781 |
| LINC01503 | ENSG00000233901 | -1.266612 | 0.00115 | LINC01503 | ENSG00000233901 | 2.233319 | 0.009119 |
| PLUT | ENSG00000247381 | -0.396611 | 0.0112 | PLUT | ENSG00000247381 | 0.287719 | 0.036626 |
| LINC01366 | ENSG00000235172 | -0.977781 | 0.00016 | LINC01366 | ENSG00000235172 | 0.966619 | 0.001171 |
| ARLNC1 | ENSG00000260896 | -1.380004 | 0.00256 | ARLNC1 | ENSG00000260896 | 1.788819 | 0.003994 |

| Table S4 List of top 20 candidates of sense PCAT5-interacting  proteins that were identified by RNA pull down and LC/MS | | | | | | |
| --- | --- | --- | --- | --- | --- | --- |
|  | | | | | | |
|  | **Accession** | **Gene Name** | **Description** | **Mass** | **Score** | **Coverage** |
| 1 | sp\|O00425\|IF2B3_HUMAN | IGF2BP3 | Insulin-like growth factor 2 mRNA-binding protein 3 OS=Homo sapiens OX=9606 GN=IGF2BP3 PE=1 SV=2 | 64008 | 1808 | 64% |
| 2 | tr\|B2R5W2\|B2R5W2_HUMAN | HNRNPC | Heterogeneous nuclear ribonucleoproteins C1/C2 OS=Homo sapiens OX=9606 GN=HNRNPC PE=1 SV=1 | 31986 | 1711 | 50% |
| 3 | tr\|H0YEG8\|H0YEG8_HUMAN | NUCB2 | Nucleobindin-2 (Fragment) OS=Homo sapiens OX=9606 GN=NUCB2 PE=1 SV=2 | 23773 | 1600 | 59% |
| 4 | tr\|B0YJ06\|B0YJ06_HUMAN | TRDMT1 | tRNA aspartic acid methyltransferase 1 variant 5 OS=Homo sapiens OX=9606 GN=TRDMT1 PE=4 SV=1 | 13159 | 1525 | 58% |
| 5 | sp\|Q01650\|LAT1_HUMAN | SLC7A5 | Large neutral amino acids transporter small subunit 1 OS=Homo sapiens OX=9606 GN=SLC7A5 PE=1 SV=2 | 55659 | 1313 | 38% |
| 6 | tr\|A0A0U1RRM4\|A0A0U1RRM4_HUMAN | PTBP1 | Polypyrimidine tract-binding protein 1 OS=Homo sapiens OX=9606 GN=PTBP1 PE=1 SV=1 | 62653 | 1102 | 53% |
| 7 | sp\|Q14126\|DSG2_HUMAN | DSG2 | Desmoglein-2 OS=Homo sapiens OX=9606 GN=DSG2 PE=1 SV=2 | 123016 | 958 | 35% |
| 8 | tr\|A0A0S2Z430\|A0A0S2Z430_HUMAN | PCK2 | Phosphoenolpyruvate carboxykinase (GTP) (Fragment) OS=Homo sapiens OX=9606 GN=PCK2 PE=2 SV=1 | 71483 | 933 | 51% |
| 9 | sp\|Q96JB5\|CK5P3_HUMAN | CDK5RAP3 | CDK5 regulatory subunit-associated protein 3 OS=Homo sapiens OX=9606 GN=CDK5RAP3 PE=1 SV=2 | 57227 | 878 | 53% |
| 10 | sp\|O76021\|RL1D1_HUMAN | RSL1D1 | Ribosomal L1 domain-containing protein 1 OS=Homo sapiens OX=9606 GN=RSL1D1 PE=1 SV=3 | 55167 | 868 | 34% |
| 11 | tr\|A0A024R3D8\|A0A024R3D8_HUMAN | DLAT | Acetyltransferase component of pyruvate dehydrogenase complex OS=Homo sapiens OX=9606 GN=DLAT PE=3 SV=1 | 69438 | 762 | 37% |
| 12 | sp\|P02533\|K1C14_HUMAN | KRT14 | Keratin, type I cytoskeletal 14 OS=Homo sapiens OX=9606 GN=KRT14 PE=1 SV=4 | 51872 | 667 | 51% |
| 13 | tr\|Q05DA4\|Q05DA4_HUMAN | P4HA2 | Procollagen-proline 4-dioxygenase OS=Homo sapiens OX=9606 GN=P4HA2 PE=2 SV=1 | 57684 | 598 | 51% |
| 14 | tr\|A0A1W2PPS1\|A0A1W2PPS1_HUMAN | HNRNPU | Heterogeneous nuclear ribonucleoprotein U OS=Homo sapiens OX=9606 GN=HNRNPU PE=1 SV=1 | 89061 | 555 | 38% |
| 15 | tr\|B0QYK0\|B0QYK0_HUMAN | EWSR1 | RNA-binding protein EWS OS=Homo sapiens OX=9606 GN=EWSR1 PE=1 SV=1 | 65174 | 511 | 26% |
| 16 | tr\|F8W930\|F8W930_HUMAN | IGF2BP2 | Insulin-like growth factor 2 mRNA-binding protein 2 OS=Homo sapiens OX=9606 GN=IGF2BP2 PE=1 SV=1 | 66859 | 478 | 44% |
| 17 | sp\|O43776\|SYNC_HUMAN | NARS1 | Asparagine--tRNA ligase, cytoplasmic OS=Homo sapiens OX=9606 GN=NARS1 PE=1 SV=1 | 63758 | 444 | 36% |
| 18 | sp\|Q15397\|PUM3_HUMAN | PUM3 | Pumilio homolog 3 OS=Homo sapiens OX=9606 GN=PUM3 PE=1 SV=3 | 73937 | 402 | 36% |
| 19 | tr\|A0A5H1ZRP4\|A0A5H1ZRP4_HUMAN | NELFB | Negative elongation factor B OS=Homo sapiens OX=9606 GN=NELFB PE=1 SV=1 | 70622 | 398 | 40% |
| 20 | tr\|A0A590UJK4\|A0A590UJK4_HUMAN | SRSF6 | Serine/arginine-rich-splicing factor 6 OS=Homo sapiens OX=9606 GN=SRSF6 PE=1 SV=1 | 26404 | 366 | 44% |

Table S5 Relationships between LIN28B expression in EC and clinicopathological parameters

| Characteristics | n | Low | | High | | High positive rate (%) | p-value |
| --- | --- | --- | --- | --- | --- | --- | --- |
| LIN28B |  | (-) | (+) | (++) | (+++) |  |  |
| Normal VS tumor |  | | | | | | <0.001 |
| Normal tissue | 49 | 28 | 17 | 3 | 1 | 8.16 |  |
| EC | 50 | 5 | 10 | 18 | 17 | 70.00 |  |

| Table S6 qRT-PCR was used to detected the expression of Lin28B mRNA  expression and analyzed the clinicopathologic characteristics of endometrial cancer  patients. | | | | |
| --- | --- | --- | --- | --- |
| clinicopathologic characteristics | | N | mean | *p* |
| Age | < 60 | 37 | 4.868 | 0.5883 |
|  | ≥ 60 | 13 | 5.401 |  |
| Clinical stage | I + II | 38 | 4.059 | <0.0001 |
|  | III + IV | 12 | 54.25 |  |
| Differentiation | High | 31 | 3.227 |  |
|  | Middle | 14 | 9.181 | <0.0001* |
|  | Low | 5 | 20.44 | 0.0003# |
| Infiltration degree | ≥ 1/2 Muscle layer | 42 | 4.533 | 0.0065 |
|  | < 1/2 Muscle layer | 8 | 17.03 |  |
| Lymphnode metastasis | Negative | 41 | 4.387 | <0.0001 |
|  | Positive | 9 | 58.35 |  |
| Distal metastasis | Negative | 45 | 4.659 | 0.0024 |
|  | Positive | 5 | 56.75 |  |
| Statistical analysis was by Mann Whitney test; * = High differentiation vs Middle differentiation; # = High differentiation vs Low differentiation. | | | | |

| Table S7 Western blot was used to detected the expression of Lin28B-Protein  expression and analyzed the clinicopathologic characteristics of endometrial cancer  patients. | | | | |
| --- | --- | --- | --- | --- |
| clinicopathologic characteristics | | N | mean | *p* |
| Age | < 60 | 37 | 2.021 | 0.2629 |
|  | ≥ 60 | 13 | 2.293 |  |
| Clinical stage | I + II | 38 | 1.628 | 0.0185 |
|  | III + IV | 12 | 2.232 |  |
| Differentiation | High | 31 | 2.215 |  |
|  | Middle | 14 | 1.929 | 0.1679* |
|  | Low | 5 | 1.467 | 0.2617# |
| Infiltration degree | ≥ 1/2 Muscle layer | 42 | 2.091 | 0.458 |
|  | < 1/2 Muscle layer | 8 | 1.83 |  |
| Lymphnode metastasis | Negative | 41 | 1.467 | 0.0345 |
|  | Positive | 9 | 2.215 |  |
| Distal metastasis | Negative | 45 | 2.193 | 0.2667 |
|  | Positive | 5 | 1.467 |  |
| Statistical analysis was by Mann Whitney test; * = High differentiation vs Middle differentiation; # = High differentiation vs Low differentiation. | | | | |

| Table S8 qRT-PCR was used to detected the expression of PCAT5  expression and analyzed the clinicopathologic characteristics of endometrial cancer  patients. | | | | |
| --- | --- | --- | --- | --- |
| clinicopathologic characteristics | | N | mean | *p* |
| Age | < 60 | 37 | 4.418 | 0.1926 |
|  | ≥ 60 | 13 | 6.543 |  |
| Clinical stage | I + II | 38 | 4.469 | <0.0001 |
|  | III + IV | 12 | 29.93 |  |
| Differentiation | High | 31 | 4.17 |  |
|  | Middle | 14 | 8.505 | 0.0001 |
|  | Low | 5 | 23.64 | 0.0017 |
| Infiltration degree | ≥ 1/2 Muscle layer | 42 | 4.547 | 0.0022 |
|  | < 1/2 Muscle layer | 8 | 17.87 |  |
| Lymphnode metastasis | Negative | 41 | 4.521 | <0.0001 |
|  | Positive | 9 | 30.41 |  |
| Distal metastasis | Negative | 45 | 4.574 | 0.0005 |
|  | Positive | 5 | 29.45 |  |
| Statistical analysis was by Mann Whitney test; * = High differentiation vs Middle differentiation; # = High differentiation vs Low differentiation. | | | | |

| Table S9 Western blot was used to detected the expression of IGF2BP3  expression and analyzed the clinicopathologic characteristics of endometrial cancer  patients. | | | | |
| --- | --- | --- | --- | --- |
| clinicopathologic characteristics | | N | mean | *p* |
| Age | < 60 | 37 | 2.27 | 0.46 |
|  | ≥ 60 | 13 | 2.11 |  |
| Clinical stage | I + II | 38 | 2.04 | 0.0003 |
|  | III + IV | 12 | 3.505 |  |
| Differentiation | High | 31 | 1.88 |  |
|  | Middle | 14 | 3.295 | 0.0007 |
|  | Low | 5 | 3.55 | 0.0313 |
| Infiltration degree | ≥ 1/2 Muscle layer | 42 | 2.26 | 0.0054 |
|  | < 1/2 Muscle layer | 8 | 3.545 |  |
| Lymphnode metastasis | Negative | 41 | 2.11 | 0.001 |
|  | Positive | 9 | 3.47 |  |
| Distal metastasis | Negative | 45 | 2.45 | 0.0185 |
|  | Positive | 5 | 3.58 |  |
| Statistical analysis was by Mann Whitney test; * = High differentiation vs Middle differentiation; # = High differentiation vs Low differentiation. | | | | |

Table S10 Relationships between IGF2BP3 expression in EC and clinicopathological parameters

| Characteristics | n | Low | | High | | High positive rate (%) | p-value |
| --- | --- | --- | --- | --- | --- | --- | --- |
| IGF2BP3 |  | (-) | (+) | (++) | (+++) |  |  |
| Normal VS tumor |  | | | | | | <0.001 |
| Normal tissue | 49 | 23 | 21 | 2 | 3 | 10.20 |  |
| EC | 50 | 3 | 4 | 21 | 22 | 86.00 |  |
