# Supplementary material for: LIN28B induced PCAT5 promotes endometrial cancer progression and glycolysis via IGF2BP3 deubiquitination
Source: Cell Death Dis. 2024 Apr 2;15(4):242. doi: 10.1038/s41419-024-06564-2 (PMC10987620; doi:10.1038/s41419-024-06564-2)
Supplement: Supplementary file 3 — Supplement Figure [file 41419_2024_6564_MOESM3_ESM.pdf]

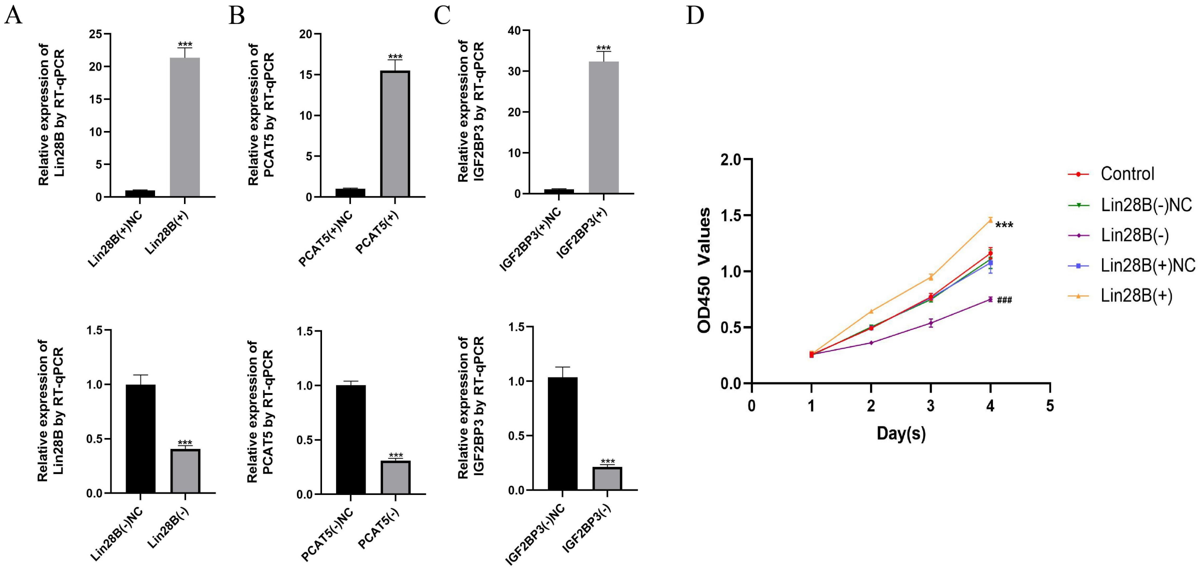

Figure S1 (A-C) Overexpression and knock-down efficiency of Lin28B,PCAT5 and IGF2BP3(n=3); (D) CCK-8 assay was used toevaluate the proliferation effect of Lin28B in ISHIKAWA(n=3).Data are expressed as means ± standard deviation. *P<0.05,**P<0.01,***P<0.001vs.Lin28B(+)NC group;$^{\#}$P<0.05, $^{\#\#}$P<0.01,and $^{\#\#\#}$P<0.001vs.Lin28B(-)NCgroup.

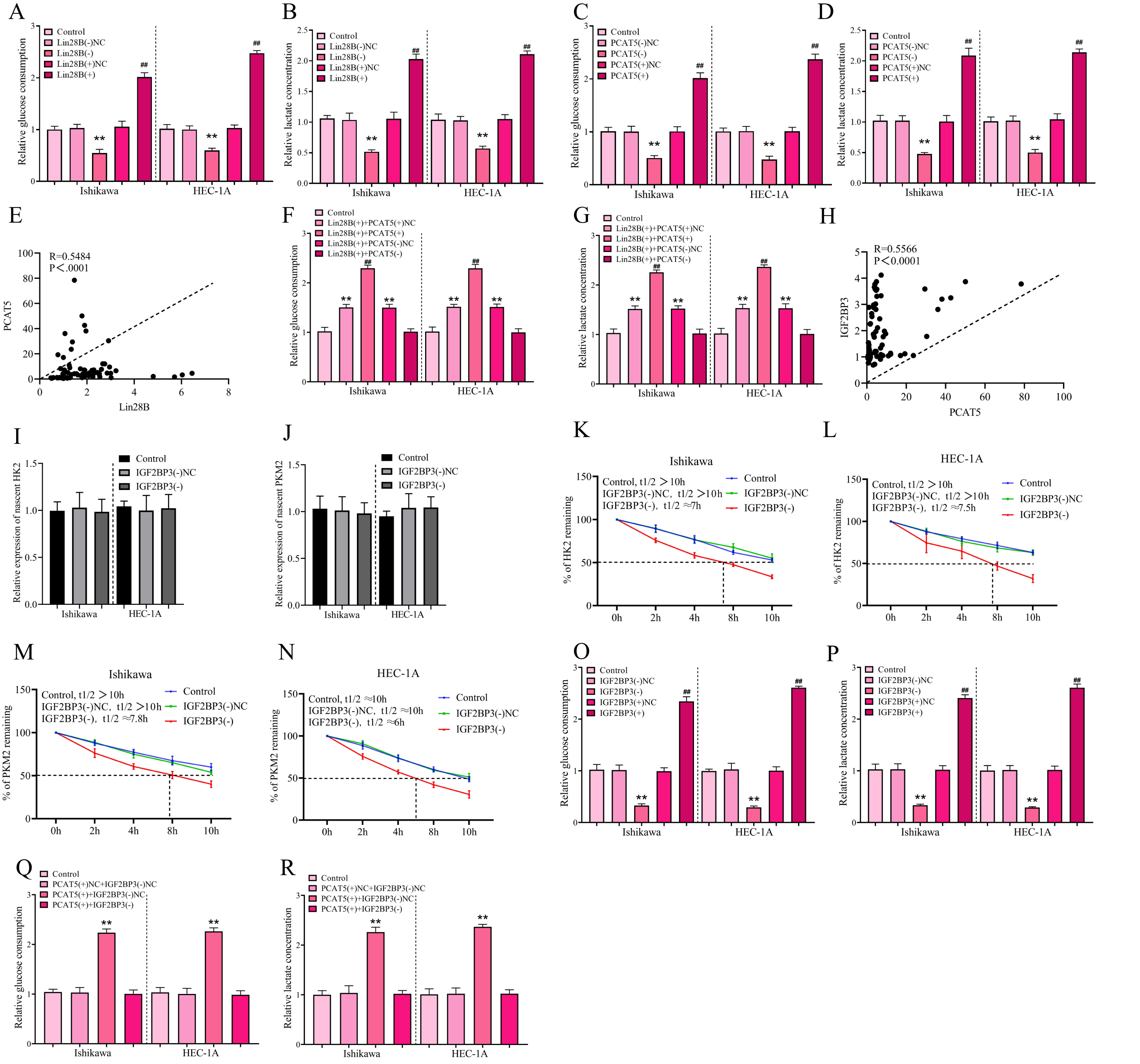

Figure S2 (A-B) The effects of LIN28B overexpression or silencing on the glycolysis ability in Ishikawa and HEC-1A cells was assessed by glucose consumption and lactate concentration assays (n=3); (C-D) The effects of PCAT5 overexpression or silencing on the glycolysis ability in Ishikawa and HEC-1A cells was assessed by glucose consumption and lactate concentration assays (n=3); (E) By Spearman r analyzing the protein expression level of LIN28B and the expression level of PCAT5 in EC tissues (n=50); (F-G) The effects of LIN28B and PCAT5 on the glycolysis ability in EC cells was assessed by glucose consumption and lactate concentration assays (n=3); (H) By Spearman r analyzing the expression level of PCAT5 and the expression level of IGF2BP3 in EC tissues (n=50); (I-J) Expression of nascent HK2 or PKM2 was measured via qRT-PCR after I GF2BP3 knockdown (n=3); (K-N) Half-life of HK2 or PKM2 was measured by RT-qPCR after actinomycin D treated in EC cells (n=3); (O-P) The effects of IGF2BP3 overexpression or silencing on the glycolysis ability in EC cells was assessed by glucose consumption and lactate concentration assays (n=3); (Q-R) The effects of PCAT5 and IGF2BP3 on the glycolysis ability in EC cells was assessed by glucose consumption and lactate concentration assays (n=3).

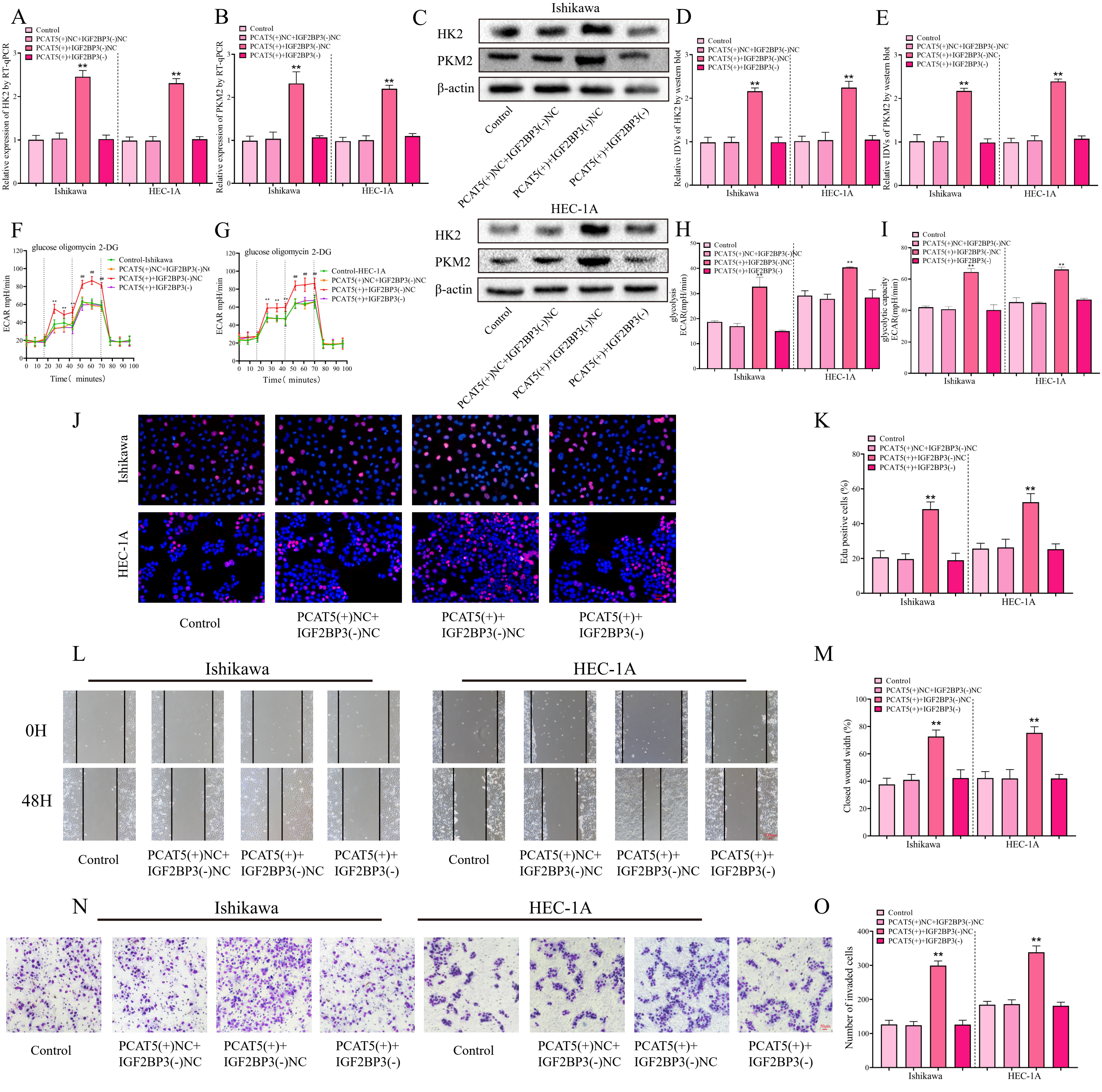

Figure S3. IGF2BP3 mediated the ability of PCAT5 to promote glycolysis, proliferation, migration and invasion of EC cells.
(A-B) Regulation of HK2 and PKM2 transcription by PCAT5 and IGF2BP3 was analyzed via RT-qPCR (n=3);
(C-E) Regulation of HK2 and PKM2 expression by PCAT5 and IGF2BP3 was analyzed via Western blot (n=3);
(F-I) The effects of PCAT5 and IGF2BP3 on the glycolysis ability in EC cells was assessed by ECAR (n=3);
(J-K) The effects of PCAT5 and IGF2BP3 on the proliferation ability in EC cells was assessed by EdU assay (n=3), Scar bar = 50μm;
(L-M) The effects of PCAT5 and IGF2BP3 on the migration ability in EC cells was assessed by wound-healing assay (n=3), Scar bar = 100μm;
(N-O) The effects of PCAT5 and IGF2BP3 on the transwell ability in EC cells was assessed by transwell assay (n=3), Scar bar = 50μm.
Data are expressed as means ± standard deviation. *p < 0.05; **p < 0.01; ***p < 0.001.

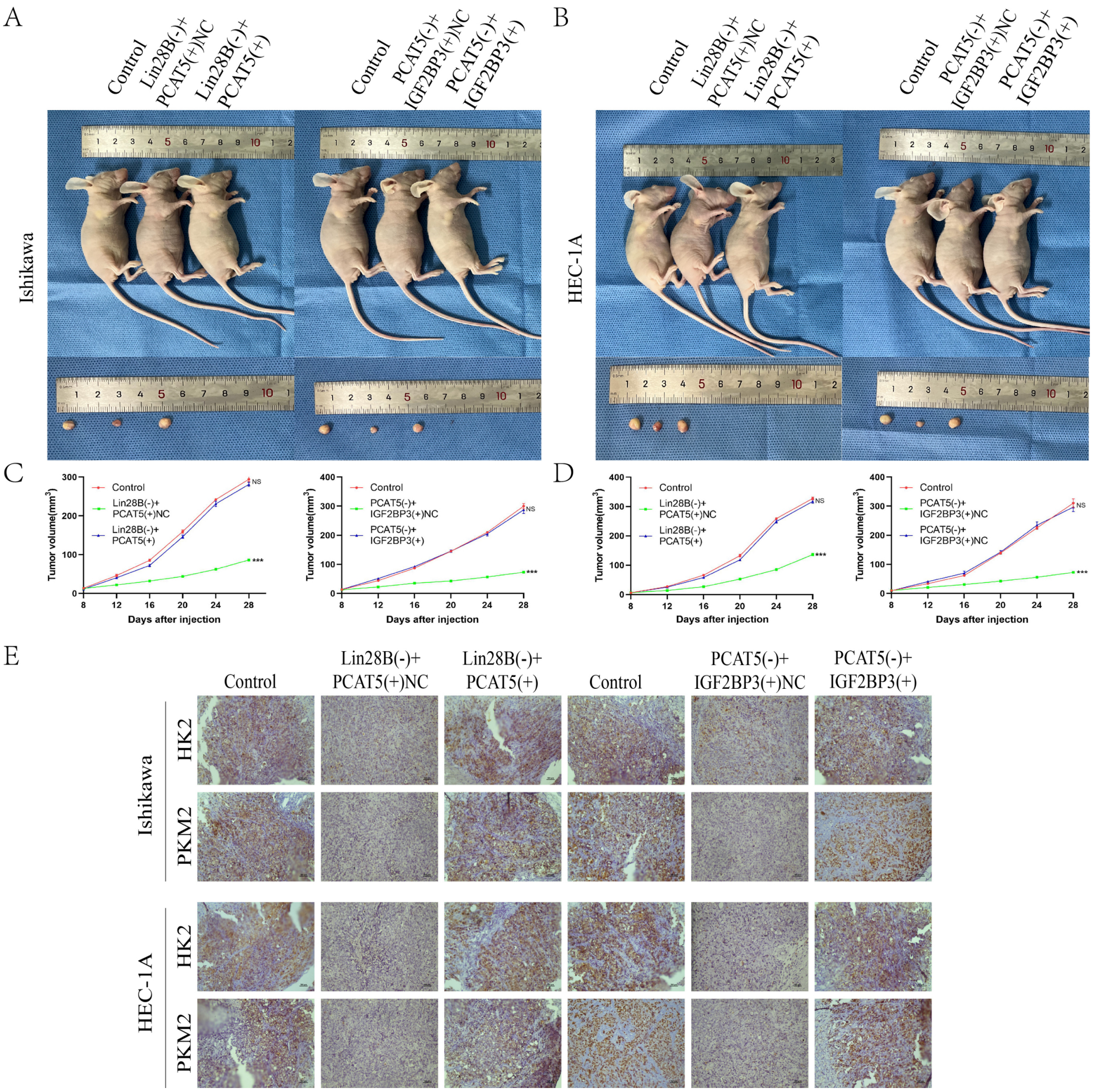

Figure S4. Rescue experiment of "LIN28B/ PCAT5/ IGF2BP3 axis" in vivo. (A-B) Subcutaneously xenografted nude mice injected with different treated cells are shown (n = 3, each group); (C-D) Tumor growth curves are shown. (E)immunohistochemical staining on the xenograft tumor from nude mice was performed to detected the effects of HK2 and PKM2 expression. Data are expressed as means ±standard deviation. *p < 0.05; **p < 0.01; ***p < 0.001.
